# Immunotherapy: an emerging modality to checkmate brain metastasis

**DOI:** 10.1186/s12943-023-01818-7

**Published:** 2023-07-15

**Authors:** Aatiya Ahmad, Parvez Khan, Asad Ur Rehman, Surinder Kumar Batra, Mohd Wasim Nasser

**Affiliations:** 1 Department of Biochemistry and Molecular Biology, University of Nebraska Medical Center, Omaha, NE-68198 USA; 2 Fred and Pamela Buffett Cancer Center, University of Nebraska Medical Center, Omaha, NE 68198 USA; 3 Eppley Institute for Research in Cancer and Allied Diseases, University of Nebraska Medical Center, Omaha, NE-68198 USA

**Keywords:** Immunotherapy, Brain metastases, Tumor microenvironment, Anticancer immunity, Immune checkpoint inhibitors

## Abstract

The diagnosis of brain metastasis (BrM) has historically been a dooming diagnosis that is nothing less than a death sentence, with few treatment options for palliation or prolonging life. Among the few treatment options available, brain radiotherapy (RT) and surgical resection have been the backbone of therapy. Within the past couple of years, immunotherapy (IT), alone and in combination with traditional treatments, has emerged as a reckoning force to combat the spread of BrM and shrink tumor burden. This review compiles recent reports describing the potential role of IT in the treatment of BrM in various cancers. It also examines the impact of the tumor microenvironment of BrM on regulating the spread of cancer and the role IT can play in mitigating that spread. Lastly, this review also focuses on the future of IT and new clinical trials pushing the boundaries of IT in BrM.

## Introduction

Metastasis to distant or multiple organs is a major obstacle to dealing with every aspect of cancer management. Some of the metastatic cases, such as brain metastasis (BrM,) further aggravate quality of life, and it is one of the primary reasons for metastasis-related deaths. An estimated 300,000 people are diagnosed with BrM in the United States every year [1]. BrM is the most common intracranial malignancy in adults and is 10 times more common compared to primary intracranial neoplasms [2]. A diagnosis of BrM carries a poor prognosis with high mortality and morbidity [3]. The median survival time depends on the primary malignancy and ranges from 4 to 16 months [4]. The most common primary sites of cancer that BrM originate from are kidney cancer (2–4%), colorectal cancer (CRC) (3–8%), melanoma (5–20%), breast cancer (BC) (15–30%), and lung cancer (LC) (40–50%) [5].

Traditionally, the mainstay of treatment in BrM has been surgical resection and radiotherapy (RT), including whole-brain radiation therapy (WBRT) and stereotactic radiosurgery (SRS) [6]. SRS, despite the name, does not involve surgical excision of tissue, but instead is a form of RT, where 3D imaging is used to deliver a highly concentrated dose of radiation directly to the tumor. The advantage of this type of RT is that it reduces toxicity to the adjacent normal tissues around the tumor and decreases the risk of radiation toxicity to healthy tissues. Chemotherapy drugs are not typically used to treat BrM because 95% of them don’t pass through the intact blood-brain barrier (BBB) [7]. Despite the use of these therapies, the mortality and morbidity rate of BrM remains high, with part of the reason being tumor heterogeneity and tumor cell plasticity. Thus, it is imperative to explore newer therapies that can cross the BBB and target different aspects of the tumor microenvironment in BrM. One such field of therapy that has gained widespread attention in the past 10 years has been immunotherapy (IT). IT has shown promising results in improving the overall survival (OS) rate in patients with BrM, both alone and in combination with traditional therapies [8–10].

ITs are the types of cancer treatments that utilize components of the immune system made by the body itself or developed in a laboratory to boost our immune system that help to identify and kill cancer cells [11]. The immune system is divided into primary and secondary immune organs [12]. The primary organs are bone marrow and thymus, while the secondary immune organs are the spleen, lymph nodes, mucosal-associated lymphoid tissues (MALTs) and gut-associated lymphoid tissues (GALTs) [12]. The bone marrow produces B cells and T cells, but T cells are primed in the thymus [12]. In addition to T/B cells, neutrophils, dendritic cells (DCs), and NK cells are other major immune cells that play an indispensable role in modulating tumor immune response. For instance, neutrophils are the key players recruited during inflammation, NK cells display a rapid and potent innate cytolytic immune response to infected cells, and DCs work as specialized antigen-presenting cells (APCs) interfacing between adaptive and innate immunity [13, 14]. T and B cells are the major components of adaptive and humoral immunity that generate an immune response according to the immunological context. CD8^+^ or cytotoxic T cells are the most widely studied and prominent anti-tumor cells that exert direct destruction through granzyme- and perforin-containing granules-mediated exocytosis. CD4^+^ T cells or T helper cells (Th-1) work through the secretion of proinflammatory cytokines that induce T-cell activation and priming, and modulate the functioning of NK cells and APCs [15]. On the other hand, CD4^+^ T cells also work as regulatory T cells (Tregs) that suppress the immune response and maintain self-tolerance. B-cells are well studied in memory-related immune response, but less explored in cancer immunology [15]. These arms of the immune system play an important role in protecting or fighting against cancer or neoplastic growth. However, the immune barrier is compromised in most cancer types or immune cells become exhausted or non-functional when encountered with cancer cells (due to the overexpression of immune inhibitory molecules such as PD-L1 in cancer cells) [16]. Therefore, to boost or activate an anticancer immune response in various cancer types, it is necessary to remove the hurdles that compromise the functioning of immune cells or components, and immunotherapeutic approaches are the ways that help to elicit anticancer immune response.

**Fig. 1:**
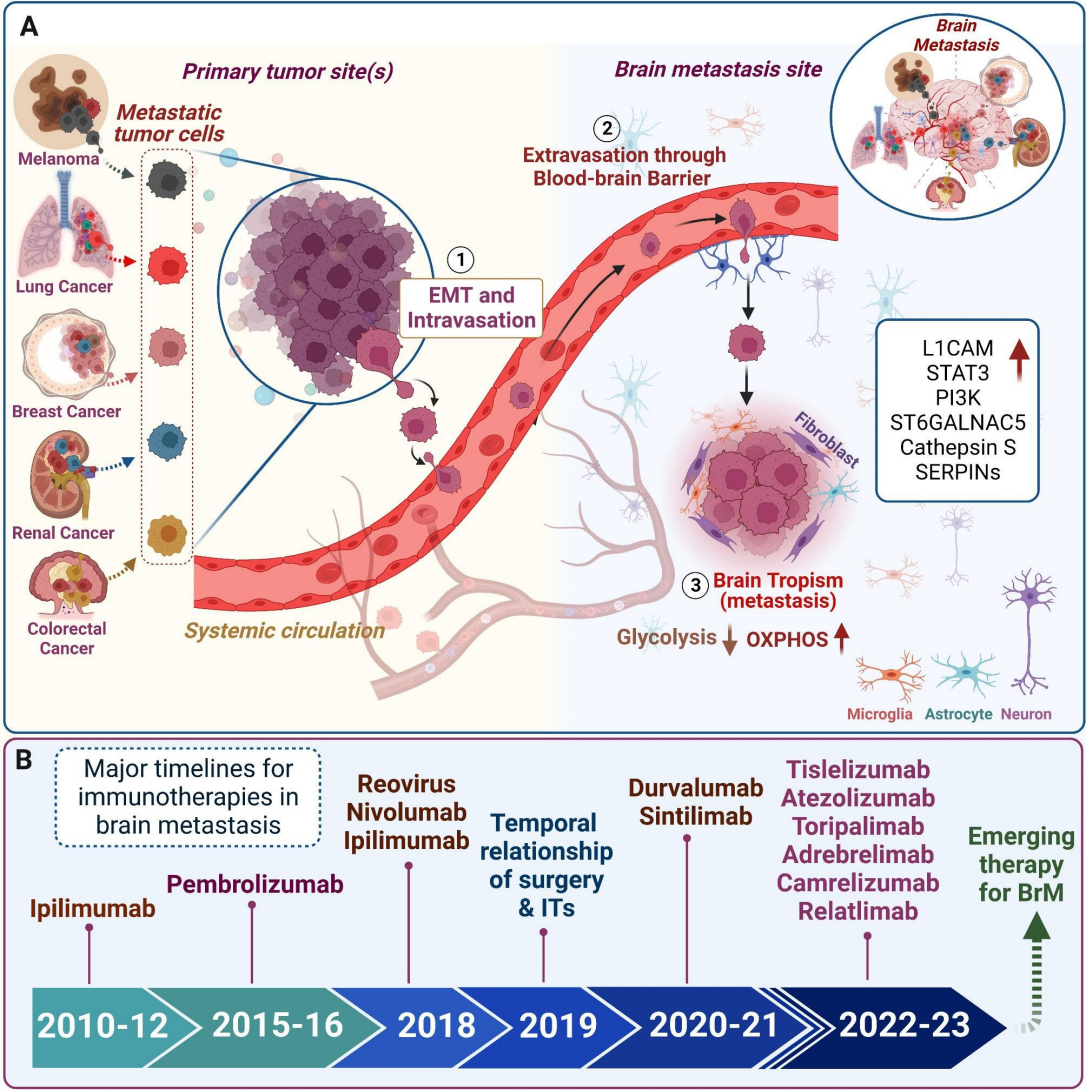
Mechanism of brain metastasis and the timeline for progress in immunotherapy. **(A)** Circulating tumor cells or metastatic cells predispose the primary melanoma, lung, breast, renal, and colorectal cancers to enter the bloodstream or systemic circulation through intravasation, reach to the brain, and extravasate through the blood-brain barrier. In the brain, these metastatic cells evade apoptosis through L1CAM mediated vascular co-option, and various other factors such as STAT3, PI3K, S6GALNAC5, and cathepsin S help these cells to colonize and grow. These cells also modify their metabolism to high OXPHOS and become less glycolytic. **(B)** Timeline progress related to the implications of various immunotherapies in treating brain metastasis.

**Fig. 2:**
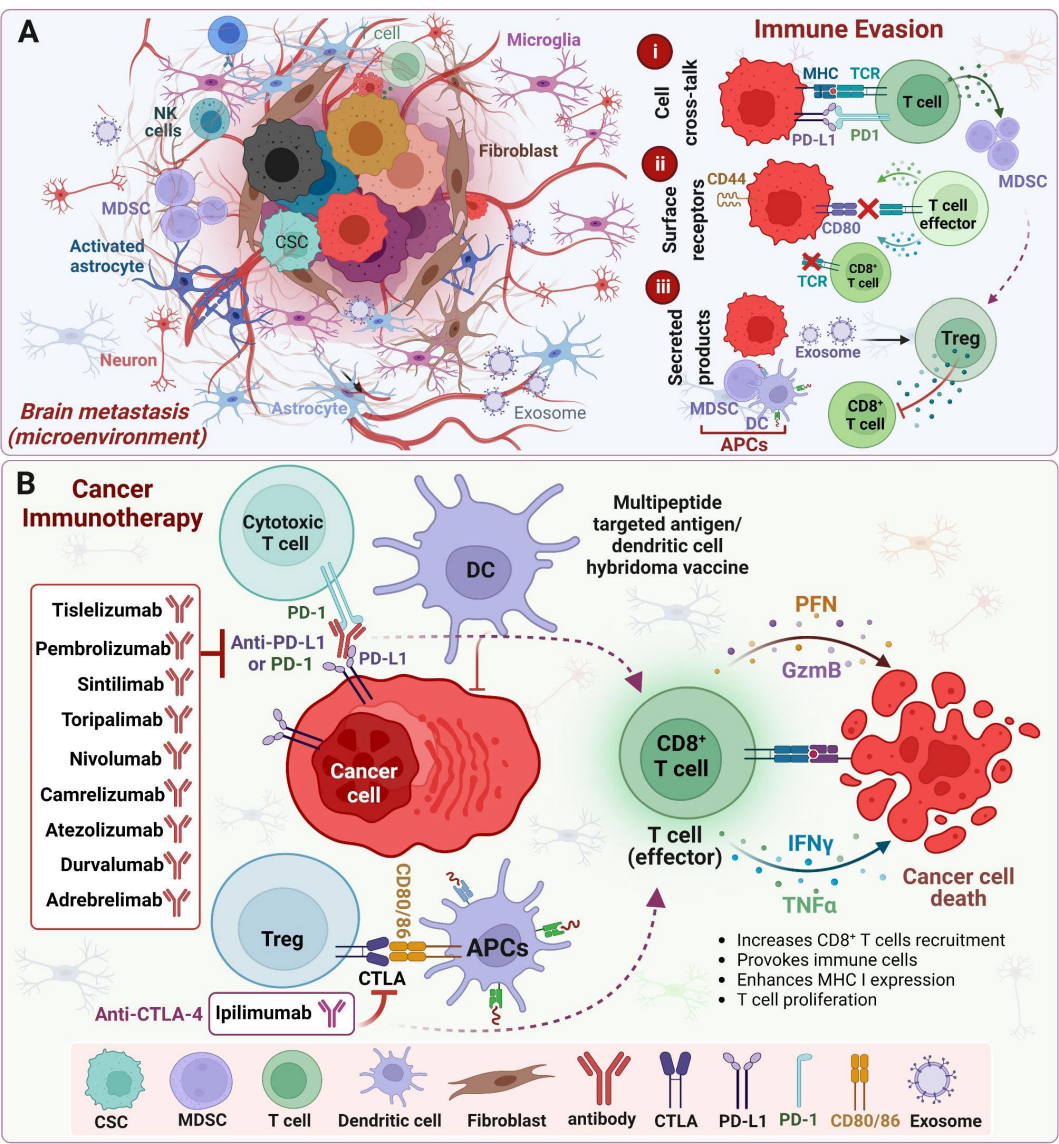
Brain metastasis and cancer immunotherapies. **(A)** In addition to metastasized tumor cells, the tumor microenvironment (TME) of brain metastasis consists of unique cell type(s), including astrocytes/activated astrocytes, microglia cells, myeloid-derived suppressor cells (MDSCs), and neurons. The neurological secretions from astrocytes/microglial cells support the growth of metastasized cancer cells in the brain microenvironment. There are multiple mechanisms for brain metastatic cancer cells to evade immune cells; it could be through upregulating the PD-L1/PD-1 axis in cancer/immune cells, overexpression of surface receptors such as CD44, or by secreting exosomes or other metabolites that enhance the recruitment of immunosuppressive regulatory T cells (Treg) cells. **(B)** Various types of immunotherapies, including anti-PD-L1/anti-PD1 and anti-CTLA4, are currently being evaluated for the treatment of brain metastasis. The ITs enhance the activity of T effector cells (CD8+) or induce tumor antigen presentation to cause immune activation, which promotes cancer cell death.

The four major ITs currently being studied in BrM include immune checkpoint inhibitors (ICIs), adoptive cellular immunotherapy, treatment vaccines, and oncolytic virus therapy [17]. ICIs block the binding of checkpoint proteins with their partner proteins, thus allowing cytotoxic T-cells to induce tumor cell death. Common checkpoint proteins that are drug targets include programmed cell death protein-1 (PD-1), programmed death-ligand 1 (PD-L1), cytotoxic T-lymphocyte-associated protein 4 (CTLA-4), and most recently, lymphocyte activation gene 3 (LAG-3) **(**Figs. 1 and 2; Tables 1 and 2**)** [18]. Adoptive cellular immunotherapy involves isolating immune cells from a patient and either expanding their numbers or using gene therapy to enhance their cancer-fighting abilities. Chimeric antigen receptor (CAR) is a synthetic receptor equipped with T-cells in CAR-T cell therapy, which allows the receptor to bind to cancer cells even if their antigens are not presented on the surface via a major histocompatibility complex (MHC). This overcomes the limitations of previous adoptive cellular immunotherapies, including tumor-infiltrating lymphocyte (TIL) and engineered T cell receptor (TCR) therapy, which requires cancer cells to present their antigens on the surface via MHC [19]. Tumor vaccines employ the use of tumor-associated antigens that are present at low levels or not present in healthy cells to prime the immune system to recognize and react to those antigens to target and kill cancer cells. Oncolytic virus therapy directly injects the virus into a tumor where it can then infect both normal adjacent tissue and cancerous cells, but only normal cells can kill the virus, whereas as cancer cells are unable to do so, causing the virus to replicate copies of itself unchecked and eventually causing the tumor cell to undergo cell death [20, 21].

**Table 1:** Immunotherapy clinical trials for the treatment of brain metastases and leptomeningeal metastases

Immunotherapy	Clinical trial identifier	Cancer type	Status	Patient Enrollment Criteria	Therapy Design	Outcome measures/outcomes
HER2.CAR T-cells	NCT03696030	HER2 + BC and BrM and/or leptomeningeal metastases	Recruiting	-18 to 75 years old -Recurrent BrM after radiation therapy (RT) or chemotherapy or untreated BrM or leptomeningeal metastases. -Karnofsky performance status (KPS) ≥70 -Life expectancy of ≥8 weeks	Intraventricular administration for 5 min, 1 dose weekly for 3 weeks	-Safety of treatment -Determine recommended phase 2 dosing -Assess disease response rate based on the Response Assessment in Neuro-Oncology Criteria (RANO), which characterizes disease as stable disease (SD), partial response (PR), or complete response (CR) in the brain -Evaluate median progression-free (mPFS) and mOS -In patients who undergo tumor resection or biopsy, analyze the TME for HER2-CAR T-cells, immune cell subsets, cytokine levels, and HER2 antigen expression levels.
Dendritic cell vaccines against HER2/HER3 with pembrolizumab	NCT04348747	Metastatic TNBC or HER2 + BC	Recruiting	-Females ≥18 years old -Not pregnant or breastfeeding -Brain lesion must meet Response Assessment in Neuro-Oncology BrM (RANO-BM) criteria -Any BrM lesion between 0.5 cm to < 3.0 cm that is asymptomatic -Radiotherapy (RT) and/or Stereotactic radiosurgery (SRS) allowed ≥2 weeks prior to first dendritic cell (DC) vaccine dose, with at least ≥1 lesion left irradiated, to use as target lesion(s).	DC vaccine anti-HER2/HER3 administered intradermally (ID) on days 1, 22, and 43	-Determine CNS response via RANO-BM -quantify BrM -Assess mPFS, mOS -Evaluate the safety of treatment
Dendritic cell vaccines pulsed with mRNA encoded tumor antigens	NCT02808416	Patients with BrM (all cancer types)	Completed	-18 to 65 years old -Undergo tumor resection or biopsy -Karnofsky scores ≥70 -No corticosteroid treatment at least one week before vaccine administration.	Biweekly mRNA-pulsed autologous dendritic cell vaccine.	-A total of 10 patients were treated with dendritic cell vaccines. -7 patients tested for anti-tumor associated antigens (TAAs), most of the TAAs induced either antigen-specific CD4 + or CD8 + T-cell responses or both -Patients treated with DC vaccines had improved OS compared to patients treated with standard therapy.
Dendritic cell vaccine	NCT03638765	BrM from BC or LC	Completed	-18 to 65 years old -Life expectancy > 12 weeks -≥1 CNS metastasis ≥10 mm per RANO-BM criteria -≥1 CNS metastasis available for reservoir placement	Injection of autologous dendritic cells intratumorally	-Evaluate the safety of treatment -Determine the feasibility of vaccine administration via Ommaya reservoir directly to tumor lesions -Ascertain tumor response, OS, neurocognitive functioning, and rate of intracranial recurrence.
Anti-ESO (Cancer/Test Antigen) mTCR (T-cell receptor) -transduced autologous peripheral blood lymphocytes and chemotherapy for treating metastases of cancer expressing NY-ESO-1	NCT02774291	HLA-A2 + BrM	Completed	-18 to 65 years old -measurable cancer that expressed NY ESO-1 as assessed by RT-PCR or IHC or serum Ab reactive with ESO -Recurrent or untreated BrM after standard treatment -≤3 brain lesions -≥8 weeks from antibody therapy including anti-CTLA-4 therapy	administration of therapy via IV over 20–30 min on day 0.	-Determine the safety and tolerability of treatment -Evaluate in vivo survival rate of T-cell receptor-engineered cells -Evaluate the objective response rate via the Response Evaluation Criteria in Solid Tumors (RECIST) criteria
Memory-like natural killer cells in combination with nivolumab and relatlimab	NCT05629546	Advanced or metastatic melanoma (including stable BrM)	Active, not yet recruiting	-≥18 years old -Eastern Cooperative Oncology Group (ECOG) Performance Status < 3	-IV infusion of memory-like natural killer cells on day 0 -relatlimab/nivolumab combination given at day 29, every 28 days for 11 cycles.	-adverse events -objective response rate -duration of response Progression-free survival -overall survival
Durvalumab	NCT04356222	Leptomeningeal metastases (LM) from NSCLC	Recruiting	-≥18 years old -pathological proof of primary NSCLC -the presence of malignant cells in CSF determined via MRI	IV infusion 1x bimonthly	-Neurological Progression Free Survival (NPFS) -overall survival -adverse events
Avelumab	NCT03719768	Leptomeningeal metastases (LM)	Active, not recruiting	-≥18 years old -life expectancy of > 8 weeks -negative pregnancy test -the presence of malignant cells in CSF or radiographic abnormalities suspecting of LM -At least 4 weeks following surgery of brain lesions	1 h IV infusion given 1x weekly for 2 weeks	-the safety of the drug, measured via adverse events -activation and number of T cells -Leptomeningeal disease response rate measured via Response Assessment in Neuro-Oncology (RANO)-Brain Metastases (BrM) criteria -OS at 3 months
Nivolumab	NCT03025256	Leptomeningeal metastases (LM)	Active, not recruiting	-≥18 years old -must have radiographic or cytological evidence of LM -must be at least 7 days out from IT, if administered	-5 min intrathecal nivolumab on day 1 of every cycle -cycle 2, patients also receive IV nivolumab for 30 min on day 1 -18 cycles, each cycle 14 days long -after 18 cycles, each cycle is 28 days long	-adverse events -OS -immunological effects of nivolumab

**Table 2:** Key immunotherapy drugs used in various cancer types to treat brain metastases

Immunotherapy Drug	Drug Class	Primary Cancer Type	References
Pembrolizumab	Anti-PD-1	NSCLC, BC(ER+), Melanoma, RCC	[1, 40, 77, 78, 85, 93, 95, 96, 119, 120, 147]
Tislelizumab	Anti-PD-1	NSCLC	[92]
Atezolizumab	Anti-PD-L1	NSCLC	[79, 111, 127]
Sintilimab	Anti-PD-1	NSCLC, SCLC	[94, 124, 127]
Toripalimab	Anti-PD-1	SCLC	[124, 127]
Adrebrelimab	Anti-PD-L1	SCLC	[126]
Durvalumab	Anti-PD-L1	SCLC	[125, 127]
Camrelizumab	Anti-PD-1	SCLC	[127]
Nivolumab	Anti-PD-1	SCLC, Melanoma, NSCLC, RCC	[40, 50, 53–55, 102, 103, 127, 170, 172, 264]
Ipilimumab	Anti-CTLA-4	Melanoma, NSCLC, RCC	[38, 40, 44, 50, 53–55, 172]
Relatlimab (only in combination with nivolumab)	Anti-LAG3	Melanoma	[41, 56, 64]

Despite being one of the leading causes of cancer-related deaths, BrM was historically understudied, and it is in the last decade we have witnessed a surge in active research focusing on BrM [22–24]. The technological advances in terms of high throughput genomics, imaging, and novel models (cell lines and animal) have helped researchers to understand BrM and develop effective therapeutic modalities such as ITs to improve outcomes in BrM patients. As IT has been emerging as a potential therapeutic strategy for BrM and various clinical trials are currently under progress **(**Table 1**)**, the compilation of recent studies and development on BrM would help researchers and clinicians gain better insight into understanding and identifying gaps in knowledge, which can further serve as a guide to improving immunotherapeutic modalities and patient outcomes. This review article summarizes the implications of immunotherapies and recent progress currently being employed in treating BrM, including in combination with SRS and chemotherapy, as well as in relation to drug resistance mechanisms and the future of immunotherapy.

## Brain metastasis formation

### Mechanism of brain metastasis

Before we delve into IT treatments currently being used, we first need to understand the main mechanism of BrM from primary tumors. BrM formation is a complex process involving numerous signaling pathways and multiple steps, thus we have limited our focus to the main mechanism of BrM common amongst primary tumors including melanoma, breast, lung, colon, and kidney cancers **(**Fig. 1A**)**. The primary tumor continuously sheds circulating tumor cells (CTCs) into the bloodstream, which not only survive, but go on to serve as seeds for secondary tumors [25]. Once CTCs reach secondary sites like the brain, they can often become dormant, but they can come out of dormancy through different mechanisms including with the help of STAT3, L1CAM, and cathepsin S [25]. A subpopulation of astrocytes are equipped with signal transducer and activator of transcription 3 (STAT3), which serves to regulate a diverse array of functions in the body [25–27]. Activation of STAT3 by brain metastatic cells via upregulation of cytokines like transforming growth factor α (TGF- α), epidermal growth factor (EGF), and macrophage migration inhibitor factor (MIF) leads to formation of astrospheres that then function to suppress CD8^+^ T cells in the brain microenvironment, allowing them to evade detection and apoptosis [25, 26]. The brain stroma contains plasmin, which can convert fas ligand (FasL), a member of the tumor necrosis factor (TNF) receptor family, which plays a role in initiating apoptosis by activating the caspase cascade, into a death signal via paracrine signaling [25]. In addition, cathepsin S functions to sever junctional adhesion molecules (JAM) like JAM-B in the BBB, thus aiding in the migration of brain metastatic cells [25]. The death signal induced by FasL against the metastatic cancer cells inhibits L1 cell adhesion molecule (L1CAM), which is needed for tumor cells to access vasculature and grow [25]. In the brain, cancer cells contain anti-plasminogen activator serpins that inhibit plasmin, thus preventing FasL from being activated and initiating apoptosis, and helping cancer cells to form brain metastasis [25].

## Healthy brain immune system versus brain metastasis

The healthy brain immune system consists of immune cells like microglia, which comprise of 80% of the immune cells in the brain, as well as myeloid cells, dendritic cells, monocytes/macrophages, natural killer cells, and B and T cells [28]. In BrM, there is a coordinated effort of cancer cells to cause immunosuppression within the brain tumor microenvironment (TME). Myeloid-derived suppressor cells (MDSCs) are alerted to the inflammation and necrosis present in the TME and work to downregulate the antitumor immune response **(**Fig. 2A**)** [29]. Furthermore, the MDSCs, in a bid to reduce inflammation, starve T cells and prevent their activation by depleting amino acids in the TME. MDSCs also produce nitric oxide (NO), which impedes IL-2 signaling, a promoter of inflammatory response, via the activation of Th1 and Th2 effector cells [30, 31]. In this environment of unchecked growth in the TME, there are reactive oxygen species (ROS) generated, which damage the healthy immune cells, including T cells. Tumor-associated macrophages (TAMs) also play a part in immunosuppression by taking on a pro-tumoral M2 phenotype, which further promotes an immunosuppressive environment [32]. DCs within the TME are inhibited by the immunosuppressive environment and remain immature [33]. Lastly, regulatory T cells are involved in creating an immunosuppressive environment by releasing multiple cytokines like IL-10, IL-35, and TGFβ, while at the same time, inhibiting co-inhibitory receptors, and devouring any IL-2 in the environment [12, 34].

## Immunotherapy in melanoma brain metastases

Melanoma is deadly cancer with a high propensity for BrM [35, 36]. The frontal and temporal lobes of the brain are the most common sites of MBrM [37]. Historically, the median OS of melanoma BrM (MBrM) patients has been poor, at 3 to 5 months, with even lower OS in patients with metastatic melanoma leptomeningeal disease at 2.9 months [35, 37–39]. Currently, the standard of care for MBrM is WBRT, SRS, a combination of the two, or IT alone or in combination with SRS. ICI drugs including ipilimumab, pembrolizumab, nivolumab, and most recently, relatlimab (in combination with nivolumab) have been approved for metastatic melanoma by the FDA **(**Table 2**)** [40, 41]. In recent years, there has been a big push to investigate whether the use of IT in MBrM also improves outcomes in patients like it has in melanoma patients.

### Biomarkers for melanoma brain metastases

To effectively treat MBrM, it is important to examine the tumor immune microenvironment (TIME) and identify predictive markers of response to IT. V-raf murine sarcoma viral oncogene homolog B1 (BRAF) is a serine/threonine protein kinase that modulates the signaling of mitogen-activated protein kinases (MAPKs), which is crucial for cell proliferation, metastasis, differentiation, and cell apoptosis. A BRAF gene mutation can lead to an alteration of the BRAF protein that regulates cell growth, leading to uninhibited and unchecked growth. BRAF mutations are found in nearly half of all patients diagnosed with melanoma, with nearly 90% of those mutations at the V600E location (glutamic acid to valine substitution) [42]. There is evidence that the use of first-line IT in patients with melanoma diagnosed before the intracranial spread of the tumor can mitigate the spread. Wang et al. examined the use of first-line IT and TT in patients with melanoma who had BRAF-mutations [43]. They found that the median BrM-free survival (BMFS) in IT-treated patients was 41.9 months versus 11.0 months for TT [43]. Furthermore, they found that the median OS showed qualitatively similar results to BMFS [43]. The patients receiving first-line anti-CTLA4 + anti-PD-1 combination IT only, or followed by second-line TT had better BMFS, improved OS, and reduced incidence of BrM [43].

One study sought to create a clinical predictive model for response and survival to ITs (anti-PD-1 monotherapy or in combination with anti-CTLA-4–ipilimumab) in metastatic melanoma patients [44]. The model for the prediction of the objective response rate in patients with advanced melanoma treated with IT presented 6 clinical parameters that included: serum lactate dehydrogenase, blood neutrophil-lymphocyte ratio, Eastern Cooperative Oncology Group (ECOG) performance status, the presence or absence of liver and lung metastases, therapy (monotherapy or combination) and type of treatment [44]. The ECOG performance status is a scale that defines a patient’s level of functioning in terms of their ability to care for themselves, their daily activity, as well as their physical ability [45]. The PFS and OS predictive models included the same parameters as above (except for the presence or absence of lung metastases) as well as the presence or absence of BrM and blood hemoglobin [44]. This study illustrates a new predictive clinical model for response to IT in metastatic melanoma that may serve as a valuable tool in clinical decision-making.

### Melanoma brain metastases treated with immunotherapy as a monotherapy

Given the success of IT in treating melanoma, there has been a push to see if IT could also be beneficial in MBrM patients. One study found that in MBrM patients treated with IT alone, the median OS was 7.29 months, a modest increase from patients not treated with RT or IT who had an OS of 3 to 5 months [35, 46]. However, the median OS in patients treated with SRS alone was higher at 9.33 months, indicating that while IT alone prolonged OS, it did not prolong it to the same level as traditional SRS [46]. This was reinforced by evidence from other studies that examined the OS of MBrM patients who received IT alone, indicating that those patients showed no objective responses and had worse outcomes than patients treated with SRS with or without IT [47, 48]. A similar study found that IT-naïve patients treated with IT alone were associated with an increased risk of death compared to IT-naïve patients treated with surgery followed by IT [49].

### Combination immunotherapies in melanoma brain metastases

**Fig. 3:**
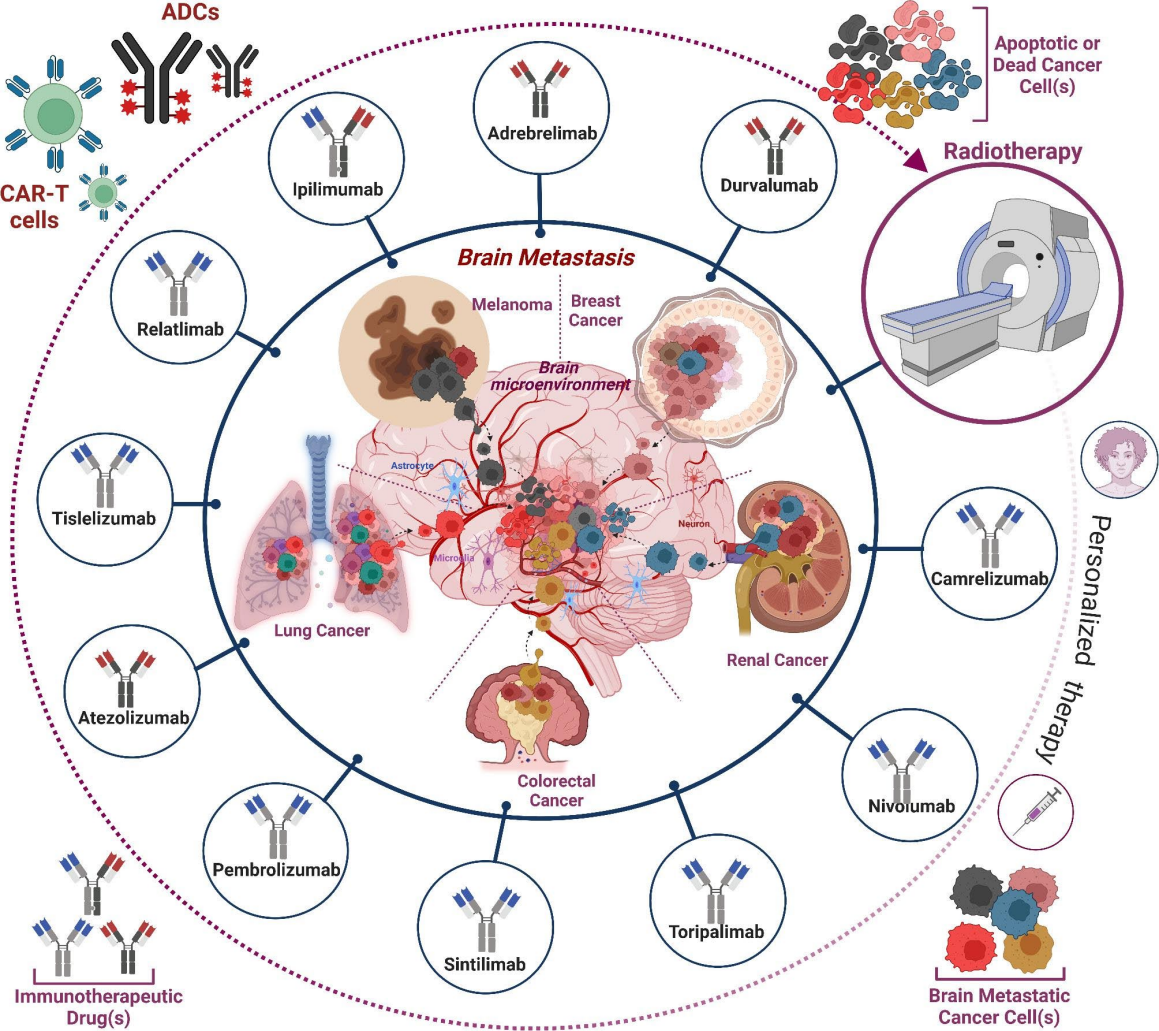
Different immunotherapies currently in clinics and their utilization for treating brain metastasis. Brain metastases occur following the migration of cancer cells from their primary tumor sites, commonly from the breast, lung, kidney/renal, colon, and melanoma to the brain. The immunotherapies displayed in the figure are in clinics for treating brain metastasis in combination with radiotherapy (RT)/ stereotactic radiosurgery (SRS). In addition to current ITs, the application of antibody-drug conjugates (ADCs) and CART cells can be harnessed to enhance the efficacies of these therapeutic modalities to treat BrM.

The addition of IT to traditional RT, like SRS has drastically improved median OS in MBrM patients. SRS in combination with immunotherapy has shown better clinical outcomes versus SRS or IT alone **(**Fig. 3**)** [8–10, 38, 50, 51]. A study found that the median OS in patients treated with SRS + IT was 15.77 months, significantly improved from the historical median OS [46]. Furthermore, SRS + IT given concurrently (defined as IT administered within 28 days of SRS), prolonged survival, with a 24-month OS rate of 47% compared to 37% for the non-concurrent group [46]. Combined concurrent IT with WBRT increased OS compared to WBRT alone, though the OS was more in the combined SRS + IT group versus WBRT + IT group [46]. The difference in OS between the two groups could also be because WBRT is used for extensive disease, while SRS is preferred for local disease, so accordingly, it is understandable why the OS in the WBRT group would be less than the SRS group. One limitation of this study is that the IT agents used were not specified and could include ICIs, interleukins, or other biomarkers. Shanker et al. conducted a similar retrospective analysis examining the concurrent use of SRS + IT versus non-current use [10]. They reported results that bolstered previous evidence that concurrent SRS + IT therapy increases the chance of achieving a complete response or partial response, with reduced likelihood of progression of the disease [10]. These outcomes definitively indicate combining IT with traditional RT increases OS for patients, and concurrent therapy is superior to nonconcurrent therapy. Furthermore, another study has found that the optimum time between SRS and administration of IT may be within 7 days, having shown a possible association with improved outcomes in patients with MBrM [52].

Traditionally, studies examining the concurrent use of IT with RT have not evaluated whether there is a difference in the outcomes based on the treatment order. One such study investigated whether the order of RT and IT affected treatment outcomes; they evaluated patients with MBrM who were split into 2 groups; one group was treated with RT first and then ICI therapy (anti-CTLA4 ipilimumab + anti-PD-1 nivolumab), while the other group was treated with ICI first and then RT [53]. Multivariate analysis revealed that there was an increase in OS and better disease control when starting with RT and then administering ICI therapy [53]. RT and 2 cycles of ipilimumab treatment administered with either RT or ipilimumab given first, showed increased frequency of activated CD4^+^ and CD8^+^ T cells with enhanced melanoma-specific T cell responses [53]. Lasso regression analysis also showed a high frequency of memory T cells and CD8^+^ T cells in the blood [53].

The use of ICIs like anti-CTLA4 and anti-PD-1 in treating MBrM patients in combination with RT is increasingly being proven to be superior in improving OS and achieving intracranial response compared to other combinations of therapies [49, 50, 54]. The CheckMate 204 phase 2 study examined the long-term outcomes of MBrM patients treated with a combination of anti-CTLA4 (ipilimumab) and anti-PD-1 (nivolumab) [55, 56]. Intracranial PFS was observed in 54.1% of asymptomatic patients and 18.9% of symptomatic patients, while the 3-year OS was 71.9% and 18.9% in asymptomatic and symptomatic patients, respectively [55]. Objectively, this data is supported by another study that found the 2-year survival rate for patients treated with anti-CTLA-4, anti-PD1, or a combination of the two was 19%, 54%, and 57%, respectively [49]. Another study evaluated patients post-GKRS and found that patients who were treated with either anti-CTLA4 or anti-PD-1 therapy or a combination of the two also showed a significantly longer OS, compared to any other types of treatment after GKRS [57]. The TT of BRAF inhibitor, mitogen-activated protein kinase (MEK) inhibitor, or tyrosine kinase inhibitors (TKI) showed no improvement in OS compared to MBrM patients who received no additional treatment post-GKRS [57]. The group further found that the occurrence of complications like hemorrhage or radiation necrosis after GKRS was not statistically significantly different in the IT/TT group [57]. This was bolstered by another study that similarly found that SRS with simultaneous IT/TT improved lesion control significantly with no noticeable difference in radiation necrosis rate [51]. Amaral et al. also investigated the use of nivolumab (anti-PD-1) combined with ipilimumab (anti-CTLA4) alone versus other combinatorial therapies in patients with symptomatic and asymptomatic MBrM [50]. In patients treated with ICI combined with SRS or surgery, there was improved OS in both symptomatic and asymptomatic MBrM patients [50]. In one study, the median OS after treatment with SRS with anti-PD-1 therapy within 3 months was 16.62 months from initial treatment with SRS, with a median PFS of 13.2 months [58]. Overall, the aforementioned studies definitively indicate that the use of ICIs in combination with SRS is vital to increasing OS in patients with MBrM. Furthermore, these studies provide strong evidence to use anti-CTLA4 and anti-PD1 drugs in combination with traditional RT should be considered as a mainstay of treatment in MBrM.

In 2022, the FDA approved the use of relatlimab, a lymphocyte activation gene 3 (LAG-3) inhibitor, in combination with nivolumab (Opdualag) for the treatment of metastatic or unresectable melanoma [41]. LAG-3 is an immune checkpoint protein found on the surface of regulatory and effector T cells, as wells as natural killer cells, B cells, and plasmacytoid dendritic cells, and works to regulate T cell activation, growth, and response, among other roles [59–63]. Approval of Opdualag by the FDA was based on the results of the clinical trial RELATIVITY-047, which examined the use of the combination drug versus nivolumab alone in 714 patients ≥12 years old, who had untreated metastatic or unresectable melanoma, including MBrM [41, 64]. The study found that the median OS of patients administered Opdualag was significantly higher at 10.1 months versus 4.6 months for the group treated with nivolumab alone [64]. The PFS was also significantly higher in the combination group at 47.7% versus 36.0% in the nivolumab monotherapy group [64].

When comparing treatment modalities used to treat MBrM, most studies have focused on the head-to-head comparison of traditional RT to IT or a combination of the two, with limited data on the comparison of chemotherapy to IT. One study compared the use of chemotherapies (carboplatin/paclitaxel, dacarbazine or temozolomide) in combination with surgery/radiosurgery (RSRS) with IT (CTLA-4 ± PD-L1 or PD-1 inhibitors) plus RSRS [65]. They found that the median OS for patients treated with IT + RSRS was 25 months, with the median OS of patients treated with chemotherapy and RSRS at 11 months [65]. Furthermore, the study also examined the use of combined TT (BRAF ± MEK inhibitors) with RSRS on median OS, finding it to be modestly improved in comparison to chemotherapy at 14 months [65].

The use of corticosteroids in concomitant treatment with IT has been shown to reduce the efficacy of IT [65]. Corticosteroids like dexamethasone are commonly used in symptomatic MBrM patients to reduce peritumoral edema [66]. Corticosteroids are known to depress the systemic immune system, so accordingly interfere with IT, which works to prime the immune system. One study showed that patients treated with corticosteroids, in addition to IT, had decreased median OS at 4 months and for patients not treated with concurrent corticosteroids had OS of 8 months [65]. Another case series examined the use of bevacizumab (a VEGF inhibitor) as a steroid-sparing agent in MBrM patients treated with IT, to see if it reduced peritumoral edema without interfering with the efficacy of IT treatment [66]. Bevacizumab was used in 12 very poor prognosis patients who either had BRAF wild-type MBrM or were resistant to BRAF/MEK inhibitor therapy [66]. All patients had previously been treated with surgery, SRS, or prior WBRT, with systemic therapy. Half of those patients had a concurrent decrease in the dose of dexamethasone, with 8 displaying reduced edema after 4 weeks of bevacizumab [66]. 10 patients received IT after bevacizumab [66]. Five patients survived for > 6 months, with one patient remaining disease-free after follow up of 4 years, without neurological deficits, despite previously being hemiplegic from BrM edema before bevacizumab therapy [66]. There were 7 patients who had adverse outcomes possibly related to bevacizumab treatment, including hypertension, intracranial hemorrhage, and gastrointestinal bleeding [66]. Bevacizumab has possibly been linked to increasing tumor-associated bleeding that might be due to an understudied mechanism regulating the regeneration of endothelial cells in the brain, leading to a compromise of blood vessel integrity [67]. MBrM patients have a high propensity for spontaneous hemorrhage independent of bevacizumab treatment, so the addition of another agent that may increase the risk of hemorrhage is concerning [68–70]. However, 10 of the 12 patients in the study displayed evidence of intracranial bleeding before beginning bevacizumab, and none of the patients displayed radiological aggravation of intratumoral bleeding that could be associated with bevacizumab [66]. Overall, this study indicates that the use of steroid-sparing agents should be considered in higher-risk MBrM patients who are going to be treated with IT, because it prolongs median OS more than corticosteroids, despite the risk of hemorrhage.

## Immunotherapy in lung cancer brain metastases

### Non-small cell lung cancer brain metastases

LCs account for the largest group of primary tumors that develop BrM [5]. It is further divided into two categories, non-small cell lung cancer (NSCLC) and small cell lung cancer (SCLC). NSCLC comprises nearly 85% of all LC cases and is further divided into adenocarcinoma, large cell carcinoma, and squamous cell carcinoma. Advanced NSCLC has a high predilection to metastasize to distant organs, including the brain [71]. Approximately 20–40% of patients diagnosed with NSCLC will go on to develop BrM, while at the time of diagnosis, 10% of patients already have BrM [72, 73]. Interestingly, different types of primary lung cancers have a propensity to metastasize to certain parts of the brain. In squamous cell carcinoma the lesions are most commonly found in the cerebellum, while in large cell carcinoma, the lesions are most often found in the occipital lobe [37, 74]. Adenocarcinoma is more diverse in its metastasis pattern, with a proclivity for both the frontal lobe and the cerebellum [74]. Traditionally, treatment modalities of NSCLC BrM have mainly included RT (WBRT & SRS) and surgical resection. SRS is the standard RT used, while WBRT is reserved for those with high tumor burden [75]. The median OS of NSCLC patients receiving traditional therapies is between 7 and 9 months [76]. Thus, it is imperative to explore new therapies that can improve the low OS.

### Biomarkers for improving the success of immunotherapy in NSCLC BrM

In recent years, studies investigating the use of ICIs have shown that they are effective treatments for various cancers. Furthermore, for advanced NSCLC without driver mutations, ICIs have become the standard first-line treatment modality [77–79]. However, studies examining the efficacy of IT in NSCLC BrM patients are limited in comparison. The efficacy of ICIs has been linked to various biomarkers in NSCLC, including TILs, tumor mutation burden (TMB), and PD-L1 expression [80]. It is important to better understand the brain immune microenvironment to evaluate how ICIs may interact with various biomarkers and either boost or decrease the efficacy of IT. One such study by Li et al. analyzed the differences in the TME of primary NSCLC tumors and paired BrM tumors and then compared them to normal lung and brain tissue samples [81]. An enrichment score represented the comparative abundance of various immune cell subtype, and it was found that the enrichment score of each immune cell subtype was decreased in BrM as compared to paired primary lung tumors [81]. Further, it was compared to normal lung and brain tissues, and they observed no significant difference in the enrichment score of immune cell types between normal and brain metastatic tissues, indicating the lower enrichment score of immune cells in BrM was tumor dependent [81]. BrM had higher fractions of CD4^+^ T-cells, dendritic cells, and neutrophils, with lower fractions of M1 macrophages and regulatory T-cells (Tregs) [81]. Tregs suppress the immune response to maintain homeostasis [82]. The differential expression analysis of ICI molecules suggests that the expression of C10orf54, otherwise known as V-domain immunoglobulin suppressor of T cell activation (VISTA), a type of transmembrane protein, and CTLA4 was decreased in BrM compared to primary lung tumors [81]. BrM were also found to have low PD-1 and CD8A expression compared to primary lung tumors [81]. Overall, these results indicate that BrM originating from lung cancer has an immunosuppressive microenvironment compared to primary tumors [81]. Traditionally, PD-1 and CTLA4 have been well-established as ICI targets, but this study indicates that the low expression of both in NSCLC BrM may decrease the effectiveness of ICIs. Additionally, PD-L1 expression can independently predict survival in patients with NSCLC BrM receiving ICI therapy [83, 84]. Interestingly, PD-L1 expression also predicts OS independent of the primary lung-graded prognostic assessment; however, the expression of PD-L1 is not associated with intracranial progression-free survival (IC-PFS) [83]. High PD-L1 expressing tumors show a robust response to anti-PD-1 (pembrolizumab) therapy, while low PD-L1 expressing tumors shows no response [85].

Kirsten rat sarcoma viral oncogene homolog (KRAS) mutations are reported to have a poorer prognosis compared to wild-type KRAS tumors [86–88]. KRAS mutations are known to lead to an upregulation of PD-L1 expression [89]. PD-L1 is upregulated by KRAS mutation through extracellular signal-regulated kinase (ERK) signaling. High PD-L1 induces the apoptosis of CD3^+^ T cells, while ICIs like anti-PD-1 antibodies or ERK inhibitors, reverse the apoptosis [89]. KRAS mutation status plays a critical role in the efficacy of ICI therapy in NSCLC BrM patients [76]. One study found that patients with KRAS mutations treated with ICI therapy within 90 days of NSCLC BrM diagnosis, of whom 97% were initially treated with SRS, had improved OS compared to those that didn’t have KRAS mutations and were treated with ICI therapy. However, patients who were treated with ICI therapy had improved OS overall, regardless of KRAS mutation status [76].

The mutations in DNA polymerase ε (POLE, a polymerase necessary for DNA replication and repair) are common in colorectal and endometrial cancer but rare in NSCLC, accounting only for 3% of NSCLC [90, 91]. POLE mutations have been linked to high PD-L1, high TMB, and infiltration of CD8^+^ T cells in the TME, all favorable factors for IT [92]. There was a case report of a patient admitted for NSCLC BrM whose tumor was detected to have a POLE mutation and TP53 mutation [92]. He was treated with combination therapy with pemetrexed, carboplatin, bevacizumab, and tislelizumab (an anti-PD1 antibody) [92]. After 4 cycles of combined therapy, the BrM had completely disappeared [92]. After 11 months of combined therapy, the patient continued to respond to ongoing therapy with no adverse events related to treatment [92]. This case provides evidence that POLE mutations correlate with higher responsiveness to IT.

Another case report described a patient with BrM from squamous cell carcinoma, who showed pathologic complete response (pCR), with continuous chemo-immunotherapy [93]. The patient was found to have negative PD-L1 expression but high TMB [93]. The patient began systemic chemo-immunotherapy (paclitaxel + carboplatin + pembrolizumab) in December 2017 and achieved a partial response following 4 cycles of therapy [93]. The patient stopped receiving any treatment after the resection of his primary lung tumor and has maintained his cancer-free status as of December 2020, three years after first starting therapy, providing evidence that high TMB may correlate to higher responsiveness to IT [93].

Epidermal growth factor (EGFR) mutation responsiveness to IT has not yet been determined, but there is limited evidence that shows that it may be a predictor for better response to IT. Nong et al. present a case report of a durable response to sintilimab (PD-1 inhibitor) in addition to chemotherapy (pemetrexed and carboplatin) in a lung adenocarcinoma patient with untreated BrM having insertion mutation in EGFR exon 20 [94]. The patient started chemo-immunotherapy in November 2019 and received 6 cycles of treatment with follow-up maintenance therapy with sintilimab and pemetrexed [94]. The patient had a PFS of 18 months as reported by the last follow-up in May 2021 and continued to receive treatment with sintilimab and pemetrexed with no evidence of toxicity [94]. Another case report by Pizarro et al. presented a similar patient with EGRF mutation in NSCLC BrM who showed a complete response to ICI plus chemotherapy after clinical response to afatinib (a tyrosine kinase inhibitor) and SRS [95]. One year after treatment, the patient remained in remission with maintenance therapy of pembrolizumab plus pemetrexed [95]. These case reports highlight the prevailing theory that EGFR mutation may have a role in boosting the efficacy of IT.

Rearranged during transfection (RET) is a proto-oncogene that becomes oncogenic upon rearrangement [96]. RET rearrangements were seen in 1–2% of NSCLC patients [96]. Nearly, 50% of NSCLC patients having a RET-rearrangement have a prevalence of developing BrM. RET-rearrangements are associated with high PD-L1 expression [96]. Riudavets et al. present a case report of a woman with initial adenocarcinoma who received chemotherapy but went on to develop BrM and liver metastases with RET-rearrangement, with high PD-L1 (90%) expression seen on tumor tissues through immunohistochemistry [96]. At that point, the patient was started on ICI (pembrolizumab) and continued that treatment for 5 cycles before it was discontinued due to liver toxicity [96]. The patient refused the proposed corticosteroid therapy but, despite no further treatment, received a normal liver test at 14 weeks, and 6 months later imaging showed a complete response [96].

Prognostic predictors of responsiveness to IT in NSCLC BrM patients are invaluable tools clinically. One study examined the prognostic, predictive value of surveying the pre-SRS leukocyte-based ratios of NSCLC BrM patients who were later treated with IT or targeted therapy (TT) [97]. Within 14 days before SRS, patients’ lymphocyte-to-monocyte, platelet-to-lymphocyte, and neutrophil-to-lymphocyte ratios were assessed [97]. Leukocyte-based ratios have been found to predict the survival of patients having BrM before treatment with SRS, without additional IT or TT [98–100]. The pre-SRS neutrophil-to-lymphocyte ratio was found to be a significant and independent factor for survival [97]. This study provides evidence that the neutrophil-to-lymphocyte ratio may serve as a relevant prognostic predictor for survival in patients later treated with IT.

### NSCLC brain metastases treatment with immune checkpoint inhibitor monotherapy

The discovery of ICI has vastly improved the OS of patients in comparison to traditional therapies and drastically changed treatment guidelines. ICI has cemented its place in metastatic NSCLC according to the National Comprehensive Cancer Network guidelines. The use of ICI monotherapy has been explored in NSCLC BrM. It shows merit in asymptomatic active BrM, with a median OS of 17.0 months and progression-free survival (PFS) of 3.19 months, which is an improvement in comparison to median OS after traditional therapies (ranging from 7 to 9 months) [76, 101].

Rounis et al. examined the efficacy of PD-1/PD-L1 inhibitor(s) as monotherapy in NSCLC BrM patients [102]. Clinical parameters such as age ≥ 70 years and no previous brain radiation therapy were associated with worse response to ICI therapy and intracranial disease progression [102]. These results expand on the findings seen in a similar trial that focused on the PD-1 inhibitor nivolumab monotherapy in non-squamous NSCLC BrM, which confirmed that while PD-1 inhibitors are active and show some benefit in OS these patients, IT monotherapy does not show the same benefit in OS as combined therapy [102, 103].

### Combination immunotherapies for NSCLC brain metastases

Tumor heterogeneity/subtype switching leads to differential responses to various anticancer therapies (monotherapies); therefore, it becomes imperative to target these tumors with a combination of two or more therapeutic modalities such as chemo-immunotherapies or a combination of more than one IT or RT in combination with IT. RT works to induce cell death and then optimize the systemic immune response induced by IT by subsequently activating and increasing T-cell infiltration [104–107]. This synergistic effort is likely the cause of the abscopal effect [108–110]. The abscopal effect is the reduction of metastatic growth at a distance from the primary site of therapy or radiation [111]. Before the widespread use of IT, the abscopal effect was rarely seen, but because of widespread IT use, it is now commonly observed across various metastatic cancer types [111]. In a lung adenocarcinoma patient with BrM, there was a reported case of extra-cranial abscopal effect following SRS, with atezolizumab as a second-line therapy [111]. The patient had pseudo-progression of the primary tumor in the lungs before remission was confirmed [111]. This case is unique because the central nervous system (CNS) is considered as immune privileged, with limited regular immune responses observed **(**Fig. 2A-B**)** [111].

Multiple clinical trials have examined the implications of SRS monotherapy versus combined SRS + ICI in NSCLC BrM patients and its effect on OS, distant brain failure, and neurological brain death [112–115]. Combined SRS + ICI therapy (anti-PD-1/anti-PD-L1 inhibitors) demonstrated decreased distant brain failure, decreased neurological brain death, and increased OS, compared to the SRS monotherapy group [112]. Two-year lesion control in the patients who received SRS + ICI therapy was 97% versus 86% for those who received SRS monotherapy [112]. Furthermore, the concomitant use of gamma knife radiosurgery (GKRS), a type of SRS, with ICIs showed increased OS without increased complications like radiation necrosis, radiation reaction, or intralesional hemorrhage [113]. Patients receiving SRS + ICI therapy also had a longer intracranial Local Progression-Free Survival (iLPFS) [114]. If ICI was administered within 7 days of SRS, there was a correlation with increased OS, compared to ICI administered greater than 7 days from SRS [114]. It is also important to note that the time interval between SRS and ICI therapy did not impact the toxicity rate [114]. The historical control group had a local failure rate of 10% in 1 year, compared to 1.1% in the concurrent IT group [115]. The addition of ICI therapy has been shown to increase the long-term OS of NSCLC BrM patients to the same level as NSCLC patients without BrM [116]. Based on these studies, ICIs have proven to be a robust treatment option for NSCLC patients with BrM in combination with traditional RT like SRS **(**Figs. 2 and 3**)**.

There is reported evidence that RT + ICI is superior to RT + chemotherapy in terms of OS in NSCLC BrM [117]. Wasilewski et al. explored the association of OS in patients with NSCLC BrM who had undergone previous neurosurgical resection and then received treatment with RT + chemotherapy or RT + ICI [117]. Patients receiving RT + chemotherapy following neurosurgery had lower OS (11.8 months) compared to patients who received RT + ICIs (23.0 months) after neurosurgery [117]. ICIs are also effective in increasing OS in patients lacking driver gene mutations in combination with RT [118]. ICI therapies in combination with RT also show a better intracranial response in NSCLC BrM patients without driver gene mutations [118].

There is reported evidence that despite stopping ICI treatment prematurely, patients continue to benefit from initial therapy and display primary tumor regression with no increase in BrM lesions [119]. Kakimoto et al. present a case report of a 69-year-old Japanese woman with giant cell carcinoma, a subtype of large cell carcinoma, with 2 brain metastases (BrMs) without any neurological symptoms [119]. The patient was initially treated with SRS for the BrM, and as the primary tumor showed high PD-L1 expression (75%), anti-PD-L1 (pembrolizumab) was administered every 3–4 weeks for 4 cycles [119]. At the end of the 4 cycles of treatment, the tumor reduced in size by 80.0% [119]. The patient experienced renal dysfunction after 4 weeks of treatment, and treatment was discontinued [119]. Twelve weeks after discontinuation of treatment, renal function was restored to normal without the use of corticosteroids [119]. Despite the discontinuation of ICI therapy, the primary lung tumor continued to regress, while the BrM remained well controlled [119]. The patient chose to undergo salvage therapy to remove the residual primary tumor in the lung, which under microscopy, revealed no tumor cells, just inflammation and residual scarring [119]. Koch et al. present a case series of 3 patients with symptomatic BrM from NSCLC [120]. The patients were treated with local ablation of BrM followed by neoadjuvant immunochemotherapy (pemetrexed, cisplatin, and pembrolizumab), and lastly, their pulmonary lesions were resected to eradicate the disease [120]. Despite treatment, one of the patients had progression of disease and passed away after 31 months of initial diagnosis [120]. At the time of submission of the paper, two of the patients remained alive and in good health with PFS and OS of 28 and 35 months, respectively [120].

While there is mounting evidence that IT is becoming the mainstay of treatment in addition to traditional RT in NSCLC BrM, there is some evidence that suggests its addition may only be useful in patients with a large volume of BrM disease. Singh et al. retrospectively examined NSCLC patients with BrM who underwent SRS followed by anti-PD-1 therapy in patients who were positive for PD-L1 antibodies and chemotherapy for those who were negative for PD-L1 antibodies [121]. The group found that the addition of anti-PD-1 therapy did not provide significant benefit for patients regarding OS or lesion response [121]. However, in lesions that were greater in volume (> 500 mm^3^), the combination of SRS with IT resulted in faster and better volumetric response [121]. They concluded that this would be particularly beneficial in patients with BrM lesions causing mass effects or lesions located in neurologically critical locations, but it may not benefit patients with few BrM lesions [121].

### Immunotherapy in small cell lung cancer brain metastases

Small cell lung cancer (SCLC) is a fast-growing and highly aggressive neuroendocrine neoplasm that accounts for 13–15% of lung cancers [71]. Approximately 10–25% of patients with SCLC have BrM at initial diagnosis, with more than 50% of patients going on to develop BrM during the course of their disease [122]. The right frontal lobe and the cerebellum are the most common parts of the brain affected by SCLC BrM [74]. The mainstay of treatment for SCLC BrM is the same as NSCLC BrM. However, despite treatment, the 12-month OS rate of SCLC remains low at 39% [123]. IT has become popular in the treatment of SCLC, with a significant increase in OS observed, but there is limited use of IT in SCLC BrM. Here we discuss literature that examines the clinical use of IT in SCLC BrM patients and their outcomes.

Huang et al. investigated the use of anti-PD-1 therapy in combination with multikinase inhibitors in a patient with SCLC BrM [124]. A female patient (59-years old) was initially diagnosed with SCLC after PET-CT and biopsy, with no evidence of BrM [124]. She underwent chemotherapy as well as thoracic RT and prophylactic brain RT. Six months after the end of RT, the patient was seen to have BrM on MRI [124]. She underwent intensity-modulated RT followed by chemotherapy [124]. Maintenance therapy included the PD-1 inhibitor sintilimab with anlotinib (a multi-targeting tyrosine kinase inhibitor). After 5.5 months, there was a failure of treatment with relapsed brain lesions [124]. A different PD-1 inhibitor, toripalimab, combined with anlotinib was started; after two cycles, the relapsed BrM completely disappeared [124]. Another seven cycles of this treatment regimen were given with sustained complete response [124]. Wu et al. present a similar case report with a patient who had stage-III SCLC and developed BrM after concurrent chemotherapy and WBRT [125]. Durvalumab, a PD-L1 antibody, was used as maintenance therapy [125]. Treatment failure occurred with the multifocal reoccurrence of BrM after the second dose of durvalumab [125]. After administration of anlotinib along with durvalumab, near complete regression of BrM was achieved, with no severe toxicity reported [125]. These case reports suggest that failure of one PD-1/PD-L1 inhibitor does not indicate failure of IT altogether and that it is still possible to continue treatment with the same or different PD-1/PD-L1 inhibitor with treatment success. The outcomes of these studies also suggest that there is a synergistic effect of the tyrosine kinase inhibitor, anlotinib, with PD-1/PD-L1 inhibitors.

Wang et al. performed a phase 3 trial comparing the effectiveness of a novel anti-PD-L1 antibody, adrebrelimab, combined with chemotherapy versus placebo + chemotherapy in extensive-stage SCLC patients, including liver and BrM [126]. It was shown that the median OS was significantly increased in the adrebrelimab group (15.3 months) versus the placebo group (12.8 months) [126]. The results of this experiment support the utilization of IT as a first-line therapy for patients with metastatic SCLC [126].

Chang et al., performed a retrospective analysis of SCLC BrM patients treated with chemotherapy and RT versus chemotherapy, RT, and at least four cycles of ICIs (PD-1 inhibitors – nivolumab, toripalimab, tislezlizumab, sintilimab, or camrelizumab and PD-L1 inhibitors – atezolizumab or durvalumab) [127]. The results showed a significant difference in the median OS, which was 13.3 months in the non-IT group and 33.4 months in the IT group [127]. The intracranial objective response rate of the IT-treated patients was more significant than the non-IT group, but the intracranial disease control rate was similar [127].

### Immunotherapy in breast cancer brain metastases

Breast cancer (BC) is the second leading cause of BrM, with up to 33% of patients developing BrM [73, 128, 129]. The most commonly affected areas of the brain by BC BrM are the cerebellum and the basal ganglia [37]. BC is traditionally classified based on the presence or absence of hormone receptors (HRs), including estrogen receptor (ER) and progesterone receptor (PR), as well as human epidermal growth factor receptor 2 (HER2). HR-positive BC subtypes account for 60-70% of all BCs and have the lowest incidence of BrM [130, 131]. Patients with HR + BC BrM have an OS of 5–10 months [132–134]. Approximately 20–30% of all BCs are HER2+, which have the highest incidence of BrM (31–50%) [130, 131, 135, 136]. If a BC is negative for ER, PR, or HER2 receptors, it is referred to as a triple negative BC (TNBC). TNBC is considered a highly aggressive cancer that proliferates rapidly and is often initially diagnosed at advanced stages. Between 22% and 50% of TNBC patients develop BrM and have 4–5 months OS of patients [130, 131, 133, 137–140]. Traditionally, treatment modalities for the treatment of BCs have depended on the receptor status of the tumor, thus, it is harder to treat a cancer like TNBC with no receptors to target. Patients with metastatic HER2 + and TNBCs have a predisposition for the tumor to metastasize to the brain compared to HR + and HER2− BCs [141].

### Biomarkers for breast cancer brain metastases

In order to effectively treat BC BrM, it is important to examine the tumor immune microenvironment and identify markers predictive of response to IT. Ogiya et al. analyzed primary breast tumors and pair-matched BrM to assess for the difference in various biomarkers [142]. There were significantly more TILs in the primary breast tumors versus the BrM tissues [142]. The primary breast tumor also featured higher CD4^+^, CD8^+^, and forkhead box P3 protein (FOXP3) positive cells [142]. In TNBC, low TIL numbers correlated with decreased OS, compared to high TIL numbers [142]. Routh et al. conducted a similar analysis of immune biomarkers in patients with TNBC. Compared to primary breast tumor tissue, BrM displayed a higher TMB, with tumor protein 53 (TP53) altered in 50% of patients [137]. Neoantigen prediction displayed high levels of endogenous retrovirus-derived MHC class I-binding peptides in both primary and BrM tumors and further predicted significantly higher single-nucleotide variant-derived peptides in BrM compared to primary tumors [137]. BrM also had decreased immune gene expression, with reduced T and B cell receptor diversity compared to pair-matched breast tumor tissue [137]. These results proved the potential of IT vaccines or ICIs in the treatment of TNBC BrM, with further scope for investigation [137].

Chehade et al. evaluated the intracranial efficacy of IT in BC BrM patients by analyzing the expression of PD-L1 in BrM tissues (PD-L1 is a predictive biomarker for IT response) [141]. In this study, positive PD-L1 expression was defined as PD-L1 ≥1% [141]. PD-L1 expression was observed in 25.0% of TNBC, 21.4% of HER2+/HR− BCs, 11.1% in HR+/HER2− BCs, and 7.1% in HER2+/HR + BCs [141]. The 24-month CNS-specific PFS was 66.7% in patients having PD-L1 expression versus 42% in PD-L1 negative BrM patients [141]. These results warrant further study into the efficacy of intracranial IT in TNBC BrM, which is paradoxically historically poorly responsive to extracranial IT, despite it having the highest PD-L1 expression among the BrM tissue samples [141, 143].

### Breast cancer brain metastases treatment with immunotherapy

Yokoi et al. examined the effectiveness of induction and activation of tumor-residing conventional type-1 dendritic cells (cDC1s), which were required for the production of CD8^+^ T cells that regress mammary tumors and potentiate the therapeutic efficacy of anti-PD1/PD-L1 in a mouse model of TNBC, which is historically poorly responsive to IT [143]. The orthotopic mammary tumors were established with subsequent BrM and then they were treated with in-situ immunomodulation (ISIM). It consisted of intratumoral injections of Fms-like tyrosine kinase receptor 3 ligand (Flt3L), which activates the proliferation of stem and progenitor cells by binding with the Flt-3 receptor to mobilize cDC1s, local irradiation, and TLR3/CD40 stimulation to activate cDC1s [143, 144]. ISIM treatment increases circulating T cells and infiltration of CD8^+^ T cells into the BrM tumors, which decreased the BrM progression and thus improved the OS [143]. Moreover, anti-PD-L1 monotherapy was inefficacious against BrM, but ISIM treatment helped overcome the anti-PD-L1 resistance, which made the tumor become responsive to anti-PD-L1 therapy, improving OS [143]. These results showcase the potential therapeutic application of IT in TNBC patients with advanced metastatic disease.

The therapy strategy for BC patients with metastatic disease remains challenging in HR + BC because of endocrine resistance, which is inevitable in ER + metastatic BCs. This is driven by ligand-independent ER reactivation, which can be modulated by gain-of-function mutations, altered interaction of ERs with their respective coactivators/corepressors, or via engagement of compensatory crosstalk among ERs, oncogenic signaling pathways, and growth factor receptors [145, 146]. Wu et al. present a case report of a patient with ER + BC with BrM, who benefited from combined anti-estrogen and IT agents, post-endocrine therapy [147]. After endocrine therapy, the patient had relapsed BC with ovarian metastatic lesions and BrM [147]. She underwent surgical resection of the ovarian lesions and then received three cycles of chemotherapy [147]. The brain lesions were unchanged in response to the chemotherapy, so chemotherapy was discontinued [147]. High T cell receptor expression was observed in the tumor, so the researchers administered the anti-estrogen drug letrozole with anti-PD-1 therapy, pembrolizumab [147]. The patient experienced a partial response and had PFS for over 21 months [147]. These results indicate that concurrent ICI therapy with anti-estrogen agents is a promising treatment option for endocrine-resistant ER + BC with BrM.

## Immunotherapy in other types of brain metastases

### Colon cancer

Colorectal cancer (CRC) is one of the leading causes of cancer-related mortality [148]. Approximately 50% of CRC patients develop metastases, with BrM representing 4–6% of all metastases cases [149, 150]. Like for other types of BrM, the traditional treatment is RT, either SRS or WBRT, and in rare cases, chemotherapy **(**Figs. 2 and 3**)**. The OS of patients with CRC BrM ranges from 2 to 15 months [151]. In the past, trials with IT to treat CRC BrM have had limited therapeutic success. Thus, there is room for new IT modalities in CRC BrM patients.

One study employed the use of a gamma retroviral replicating vector that encodes for a cytosine deaminase (CD), Toca 511, to selectively infect malignant CRC BrM tumor model cells [152]. The encoded CD protein was expressed in infected cells and converted the administered an oral anti-fungal prodrug, 5-fluorocytosine (5-FC) to an anti-cancer drug, 5-fluorouracil (5-FU). This system allows higher levels of 5-FU to be generated directly at the site of the tumor than systemic administration of 5-FU [152]. Toca 511 and 5-FU have been found to have immunotherapeutic effects that target myeloid-derived suppressor cells (MDSCs) [152]. MDSCs are attracted via tumor-mediated signals and subsequently mature in the TME [153–156]. Some studies have shown that MDSCs modulate cancer cell immune evasion via suppressing the immune anti-tumor response through multiple mechanisms [157]. 5-FU has been shown to deplete MDSCs, thus the use of Toca 511 and 5-FC would theoretically produce a higher local dose of 5-FU in the TME, depleting immune-suppressive cells and boosting the tumor immune system [152, 158]. The study found that the combined use of 5-FC and Toca 511 had significantly decreased MDSCs in CRC tumors in the brain and liver [152].

### Kidney cancer

In renal cell carcinoma (RCC), BrM occurs in 8–16% of advanced cases and is associated with a median OS of 5 to 13 months [159–164]. The OS of patients has improved within the past decade as TT, such as tyrosine kinase (VEGF receptor) and rapamycin inhibitors, have demonstrated PFS, along with more aggressive use of local BrM therapy [165–169]. Despite the advances in IT, surgery and radiation remain the cornerstones of treatment. However, there is a drive to discover other therapeutic modalities that can diminish disease and prolong survival.

IT has demonstrated some efficacy in RCC BrM in recent years; a retrospective study examining the impact of nivolumab monotherapy in RCC patients having BrM demonstrated a limited intracranial response rate of 12% to treatment [170]. Combined IT and SRS treatment in RCC BrM has shown more success, such as in the retrospective analysis conducted by Uezono et al., where they determined that the median OS was considerably higher in the SRS & IT group at 27.2 months compared to 14.9 months in the SRS monotherapy group [171]. A decreased dose of ionizing radiation (2 Gy decrease) used in the combined SRS & IT group demonstrated the same efficacy in lesion control of BrM compared to the SRS group, with no increased risk of CNS toxicity [171]. Chen et al. further examined whether the timing of ICI affected the outcome. The study investigated the efficacy of concurrent SRS & ICI (ipilimumab, nivolumab, and pembrolizumab) versus nonconcurrent SRS & ICI versus SRS alone in patients with NSCLC BrM, MBrM, and RCC BrM [172]. The study parameters defined concurrent ICI as ICI given within 2 weeks of SRS [172]. Chen et al. observed that concurrent SRS & ICI predicted a decreased propensity for developing 3 or more new BrMs [172]. The median OS for concurrent SRS & ICI was by far the most at 24.7 months, compared to 12.9 months and 14.5 months for the SRS monotherapy and nonconcurrent SRS & ICI therapy group, respectively [172]. These studies demonstrate that IT has established itself as a cog in the wheel of treatment for RCC BrM with superior response in combined therapy with SRS versus monotherapy. Further development of new ITs is needed to continue to test the boundaries of treatment **(**Figs. 1 and 3**)**.

## Alternative immunotherapies in brain metastases treatment

CAR-T cell-based immunotherapy has shown marked benefits in hematologic malignancies like leukemia and lymphoma [173–176]. However, there has been limited success in developing CAR-T cell therapeutic models for solid tumors. One study reported the use of CAR-T cells targeting B7 homolog 3 protein (B7-H3), an immune checkpoint molecule, in vivo in xenotransplant models of orthotopic and metastatic NSCLC [177]. B7-H3, also known as CD 276, is highly expressed in tumor cells [178]. In normal human tissues, B7-H3 mRNA is widely expressed; however, a restricted expression of B7-H3 protein has been reported [177]. High expression of B7-H3 has been implicated in lowered survival, increased recurrence rate, and decreased prognosis in various cancers, including lung cancer, breast cancer, and brain tumors, among others [179–182]. It has been found that CAR-T cells can be engineered to migrate toward tumors by using chemokine gradients; this method can also be used to overcome the BBB [177, 183]. CCL2 is the most highly expressed gene in NSCLC and BrM that codes for the CCL2 chemokine [177, 184]. The C-C chemokine receptor type 2 (CCR2) is present on T cells and is the receptor for the CCL2 chemokine. The group leveraged the CCL2/CCR2 axis by engineering T cells that overexpressed the CCR2b receptor (an isoform of CCR2, which caused superior migration of T cells towards the CCL2 gradient). The group constructed a bicistronic vector encoding B7-H3.CAR and CCR2b. The study found that the co-expression of CCRb and B7-H3 in CAR-T cells significantly improved the capability of the T-cells to pass the BBB, subsequently augmenting antitumor activity in mouse models of NSCLC BrM lesions [177]. This indicates that there is scope to perhaps utilize T-cell chemotaxis to treat NSCLC BrM patients in the future.

A preliminary study reported the use of CAR T-cell-based IT in HER2 BC BrM in xenograft mouse models [185]. The HER2-CAR T-cells were optimized with protein cluster of differentiation 28 (CD28) or receptor tumor necrosis factor ligand superfamily member 9 (4-1BB), and then functional activity was evaluated through cytokine measurement, T-cell proliferation, and tumor-killing capability [185]. HER2-CARs with the 4-1BB costimulatory domain were better at targeting tumors, had decreased T-cell exhaustion, and augmented proliferation capacity in comparison to HER2.CARs with the costimulatory CD28 domain [185]. Robust in vivo antitumor activity was observed in the treatment of multifocal BrM after intraventricular delivery of HER2.CARS T cells in mouse models [185]. This rudimentary study provides reasoning for the development of CAR T-cells in the treatment of BC BrM in humans in the future.

The use of dendritic cells in IT has gained some popularity, with novel approaches to the treatment of BrM. One such therapy is adoptive T-cell transfer (ATCT) therapy, which uses recombinant adeno-associated viruses transfected dendritic cells that encode 1 or more tumor-specific antigenic determinant genes, which in turn activate T lymphocytes to generate cytotoxic lymphocytes (CTLs) [186–188]. The CTLs are equipped to lyse tumor-associated antigen (TAA)-positive malignant cells [189]. A preliminary study reported the use of ATCT therapy in 3 NSCLC (adenocarcinoma) patients with BrM in China [189]. The study used TAAs, carcinoembryonic antigen, and prostate-specific membrane antigen as targets of ATCT treatment because these antigens were expressed in the tumor tissues of all 3 patients [189]. The patients received 4 cycles of ATCT treatment (once every 2 weeks) [189]. MRI of the brain was done 1 month after ATCT infusion treatment, which showed BrM had completely disappeared [189]. Of the 3 patients, 2 were living at the end of the study, with respective OS of 40 and 25.3 months, while 1 passed away and had an OS of 26.7 months [189]. The surface expression of CD69 on T lymphocytes is an early marker of activation, the expression of which is seen to be upregulated after the activation of T lymphocytes [189–191]. Upon analysis of the patient’s T lymphocytes after each infusion, there was a clear trend of increasing the percentage of CD69^+^ CD8^+^ cells [189]. This rudimentary study invites room for further research into the use of ATCT therapy in patients with NSCLC BrM.

Dendritic cells have also been employed in MBrM treatment. One case report describes the use of autologous tumor-lysate-loaded dendritic cells (TL-DC) injected intradermally in the treatment of MBM in a patient [192]. The 44-year-old MBrM patient was initially treated with GKRS for her BrM [192]. She then also received four injections of Melanoma-associated antigen 3 (MAGE-A3 - a well-known cancer-testis antigen, a group of antigens that are originally expressed in male germ cells in adults but in cancer are common tumor antigens), as part of a clinical study LUD01-006 [192]. Unfortunately, because of rapid progression, the patient was removed from the study and was subsequently treated with autologous TL-DCs on a compassionate-use basis [192]. The patient has continued to routinely receive TL-DCs injected once a month, for a total of 133 vaccines up until the submission of the case report [192]. The patient remains in complete remission 10 years after combined treatment [192]. This remarkable case indicates that despite a grim prognosis and recurrence of cancer, IT in the form of dendritic cell injections can eradicate BrM in conjunction with combined RT.

Dendritic cell injection has also been used in metastatic RCC as an immune enhancer. The basis of this treatment was cross-priming, where infected dendritic cells initially secrete inflammatory factors that activate and recruit non-infected bystander dendritic cells [193]. The group synthesized a cellular adjuvant that contained pre-activated dendritic cells that produced high levels of dendritic cell activating and recruiting factors [193]. The dendritic cells were injected directly into the renal tumor twice at a two-week interval, with a planned nephrectomy. This clinical study of 12 patients initially excluded patients with BrM, but one patient developed BrM during follow-up treatment [193]. The patient with BrM, after treatment with the dendritic cell injection and subsequent initiation of sunitinib (multiple receptor tyrosine kinases inhibitor) treatments, notably had complete disappearance of all four brain lesions, with continued response to treatment 38 months after [193]. This indicates that in the near future, pro-inflammatory allogeneic dendritic cells can be extracted from healthy blood donors and later deep-frozen to be used in patients as immune enhancers.

## Brain microenvironment in the modulation of immunotherapy response in primary brain neoplasm versus brain metastases

Recent research on the BrM-induced tumor microenvironment interrogation revealed a distinct population of immune cells, including infiltrating macrophages, tissue-resident microglia, T-cells, and neutrophils **(**Fig. 2A**)** [194–196]. In both primary brain neoplasms, like glioblastoma and glioma, and BrM, underlying biological processes enhance tumorigenicity and metastases. BrM and primary brain malignancies differ in the cell composition of the TME, which can influence the IT efficacy. BrM have a higher number of lymphocytes and neutrophils, while primary brain neoplasms like gliomas have a higher number of myeloid cells [194]. Gliomas are classified based on the mutational status of isocitrate dehydrogenase (IDH), which may act as an inhibitor of T cell activation when mutated by producing oncometabolite 2-hydroxyglutarate [197]. Tumor-associated macrophages, including monocyte-derived macrophages and tissue-resident microglia, are found in abundance in gliomas, while T cell numbers were lower, especially in IDH mutant tumors, in comparison to BrM [194]. This provides evidence that gliomas are immunologically cold tumors, meaning they are unlikely to trigger a strong response by the immune system, even if primed by immunotherapy since they contain few T cell numbers [198].

**Fig. 4:**
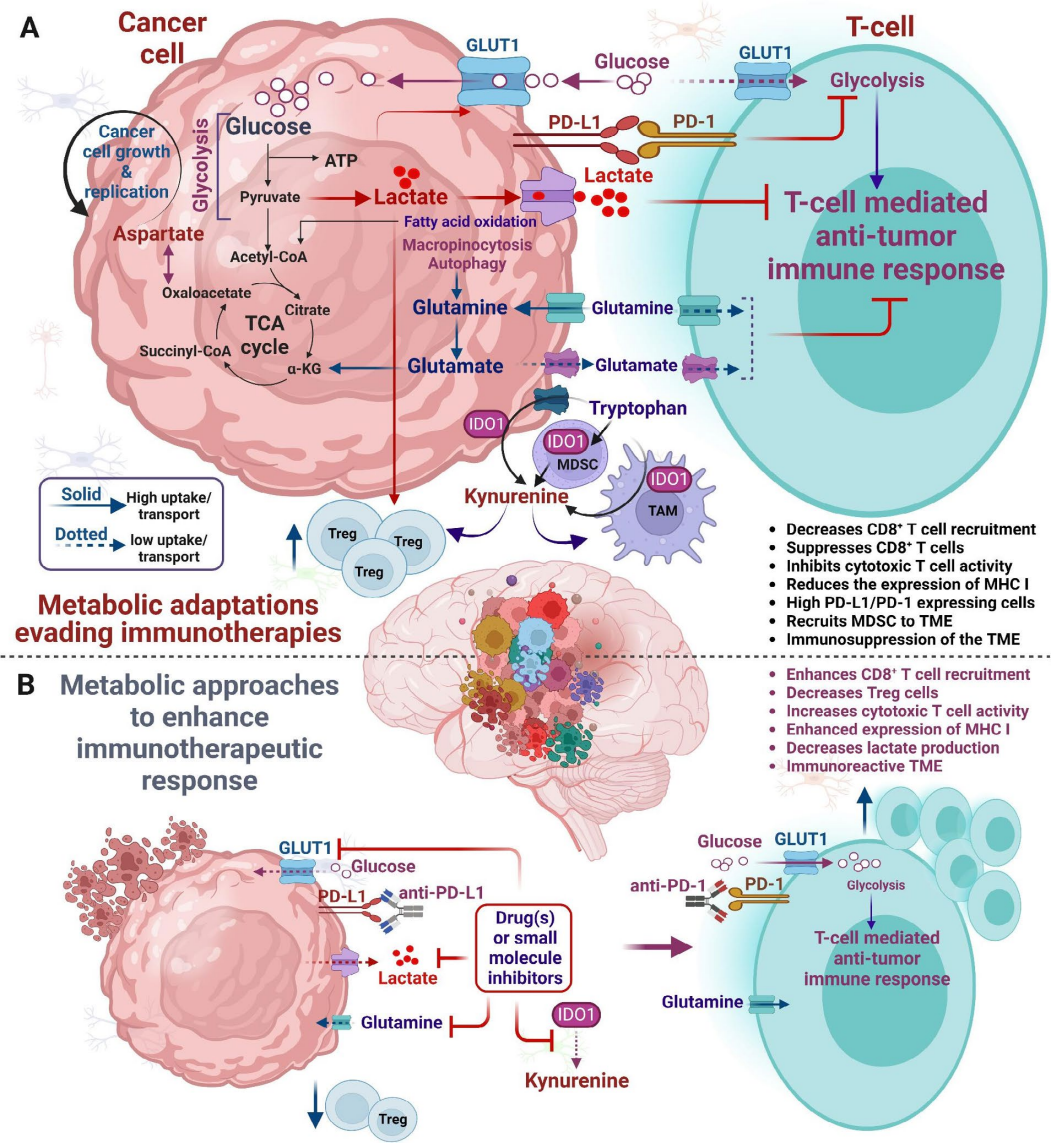
Metabolic milieu of tumor microenvironment and its impact on the functioning of immunotherapies or T cells. **(A)** Metabolic struggle and/or metabolic reprogramming transpire between various tumor-infiltrating immune cells and tumor cells. Both T cells and tumor cells preferentially utilize glucose to meet their energy demands. Due to high proliferation potential and high energy needs, tumor cells metabolize most of the glucose through aerobic glycolysis and produce high levels of lactic acid (lactate) in the TME, thereby, decreasing glucose availability for immune cells. The lactate-enriched and glucose-deprived TME impairs T cell functioning, recruits more (regulatory T cells) Tregs, and polarizes microglial cells towards a pro-tumorigenic phenotype and tumor-associated macrophages (TAMs). There is further competition for amino acids, including glutamine/glutamate and tryptophan between T cells, myeloid-derived suppressor cells (MDSCs), and tumor cells. The availability of these amino acids in the TME that modulate T cell-mediated immune response, such as kynurenine (a product of tryptophan catabolism) produced by indoleamine 2,3-dioxygenase 1 (IDO1) present in tumor cells, MDSCs, and TAMs blocks activation of T cells and promotes the recruitment and production of immunosuppressive Treg cells. The brain microenvironment has high glutamine and tryptophan; therefore, tumor cells easily adapt to the brain microenvironment utilizes these amino acids for their growth and development. Lactate production in the TME also increases the expression of PD-1 on T cells and PD-L1 on tumor cells and suppresses the activity of immune cells. **(B)** The immunotherapeutic response of ITs could be enhanced by targeting the various metabolic regulators of tumor cells and immune cells. The utilization of inhibitors that specifically target glucose transporters (GLUT1), lactate production, IDO1 activity, and glutamine utilization in tumor cells could be a potential therapeutic strategy to enhance the efficacies of ITs in brain metastasis.

Cancer as well as immune cell metabolism are key factors for determining the functioning and efficacies of ITs [199, 200]. It is widely accepted that the tumor cells’ aberrant metabolic requirements lead to a nutrient scavenged microenvironment, which eventually challenges the activation and proper functioning of immune cells **(**Fig. 4**)** [199]. Thus, it is imperative to characterize the metabolic requirements of various immune cell subsets. It will enhance understanding the essential role of TME in the functioning of immune cells and simultaneously leverage the needed information to design therapeutic interventions. Nutrient availability is unique in various organs; therefore, metastatic cancer cells migrating to the brain need to reorganize their metabolic nodes to come in parallel with the metabolic flux at the metastatic niche [201]. Lipids, serine, and glycine are some scarce nutrients that compel cancer cells to make them de-novo [202, 203]. The availability of immune cells surrounding BrM allows for anti-tumor activity, but the struggle for nutrients with metabolically enhanced tumor cells sheds misfortune on immune cell metabolism for their anti-tumor activity **(**Fig. 4**)**. CD4^+^ T-cells infiltration is high in lung cancer BrM and CD8^+^ T cells are high in melanoma-derived BrM. However, the status of CD4^+^ T cells is anergic, and CD8^+^ T cells is exhaustive and have PD-1 upregulated. Treg cells have also been characterized in BrM, but they are considerably low in melanoma BrM compared to breast and lung cancer BrM [194]. The energy source for T cells in early effector function, cytokine production, proliferation, and mitochondrial biogenesis include glucose, glutamine, acetate, glutamate, arginine, and serine [194, 204–211]. Methionine also helps in regulating the Th17 inflammatory potential and protein synthesis in T cells [212, 213]. Fatty acids, as well as tryptophan, kynurenine, and itaconate, are also among some of the metabolites that are involved in the proliferation of the T-cells production of proinflammatory cytokines and also aid in the suppression of Treg cells in the TME [214–220]. Serine, glycine, and fatty acids are limited in the brain TME for cancer cells; thus, they must reprogram their metabolism so that they either make these metabolites de-novo or use alternate pathways that are independent of these scarce metabolites [202, 203]. Both glucose and glutamine are plentiful in the brain, but these metabolites are rapidly used by tumor cells; thus, T cells adopt different metabolic pathways for sustenance, but this eventually leads to either an erroneous immune response or serves to completely dampen it [221]. BrM cells are highly glycolytic; thus, lactate production is another reason for immunosurveillance failure leading to overt metastasis [222, 223]. Interestingly, when in-vivo TILs were isolated and analyzed for the effector function, it was found to be poor. In these T cells oxidative phosphorylation (OXPHOS) was inhibited, and loss of peroxisome proliferator-activated receptor gamma coactivator 1-alpha (PGC1α), was linked to modulating the inhibition of OXPHOS. Therefore, upon ectopic expression of PGC1α, the cytotoxic activity of the TIL was restored, implying that OXPHOS could also play a major role in immunity [224]. Another aspect that could play a role in BrM is mitochondrial dynamics, where aberrant fission and fusion could also dampen the immunity, however, this has yet to be explored in context of BrM [225]. Since inflammation drives BrM and succinate is a metabolite that modulates the inflammatory pathway, there is cause to believe that interference caused by the inhibition of various complexes of the electron transport chain can yield targets that could eventually enhance the therapeutic response of the immune checkpoint blockade [226]. To enhance ICIs, dietary formulations are now aggressively being tried in different cancer settings [221]. It has been found that hypocaloric, ketogenic, and low protein isocaloric diets are correlated with increased TIL, corroborating with ITs to target malignancies [221].

## Leptomeningeal metastasis: a clinically important entity of brain metastases

Leptomeningeal metastasis (LM) is a distinct and clinically relevant subset of BrM that is defined by cancer cell infiltration into the cerebrospinal fluid (CSF), arachnoid space, subarachnoid space, and pia mater [227, 228]. There are four possible routes of LM including spread from BrM, hematogenous invasion, spread from spinal and cranial nerves, and metastasis from the perivascular lymphatic pathway [229]. LM is diagnosed in 10% of patients with metastatic disease from solid tumors [230–233]. The primary cancers associated with the highest LM are BC (11–64%), followed by LC (14–29%), and melanoma (6–18%) [234]. BC accounts for most cases of LM because of its high incidence, with 2.26 million new cases worldwide every year, according to cancer statistics by GLOBOCAN [235]. Historically LM patients have low survival, ranging from 4 weeks to 6 weeks if untreated, with median OS reaching 3 to 6 months after systemic therapies [231, 236]. The traditional treatment therapies for LM include WBRT, intrathecal treatment (via Ommaya reservoir or lumbar puncture), and tyrosine kinase inhibitors [237].

IT to treat LM is an emerging therapy that has shown modest success. A phase 2 trial using pembrolizumab (anti-PD-1) in patients with LM from primary BC and NSCLC, among other cancers, showed an improved median OS of 3.6 months, exceeding the primary endpoint goal of 3 months [238]. All patients received prior systemic therapies, including RT or surgery [238]. Of the 20 patients who underwent pembrolizumab therapy, 12 were alive at 3 months after initial enrollment [238]. A similar phase 2 trial also examined the safety and efficacy of pembrolizumab in LM patients and found a modest improvement in median CNS progression-free survival of 2.9 months and median OS of 4.9 months, with a CNS response rate of 38% [239]. Another phase 2 trial examining the efficacy of combined ipilimumab and nivolumab in LM found that this treatment regimen was effective in prolonging median OS to at least 3 months, with 8 out of 18 patients surviving until that endpoint [240]. The median follow-up was 8.0 months, based on patients that were still alive [240]. These clinical trials show that while IT does increase median OS in LM compared to untreated LM, there is not a marked difference in OS between patients treated with IT after systemic therapies versus traditional treatment alone. This can partially be attributed to the lack of clinical trials of IT used in LM patients, as well as perhaps, the application of treatment. A phase 1 trial (NCT03025256), with interim results published, showed that intrathecal administration of nivolumab, with the addition of subsequent intravenous administration in patients with LM originating from melanoma had a median OS of 4.9 months [241, 242]. This unique approach of dual administration of therapy shows promising results, with ongoing accrual of more patients, including patients with LM from lung cancer [241, 242]. Overall, the use of IT in LM so far does not display the same robust response seen in BrM. One reason for the lack of robust response of LM to IT compared to BrM could be due to the differences in the immune microenvironment. Within the leptomeninges, T-lymphocytes are immunologically disparate from the T-lymphocytes found in pair-matched BrMs [243]. They express an enhanced immunosuppressive phenotype [243]. Furthermore, the CSF contains dysfunctional CD8^+^ T cells and CD4^+^ T cells that do not adequately respond to ICI therapy [243, 244].

There are ongoing clinical trials that are further pushing the boundaries of IT in LM patients. One such trial is NCT04356222, which is examining the efficacy of durvalumab (anti-PD-L1) in combination with intrathecal chemotherapy in NSCLC LM patients. Another clinical trial, NCT03719768, is employing the use of avelumab, an FDA-approved IT for metastatic Merkel cell carcinoma and advanced urothelial carcinoma, in combination with WBRT **(**Table 1**)**.

## Thought-provoking facts for drug resistance, immunotherapy, and brain metastases

The brain is a protected organ for hiding the metastatic cells that evade systemic therapies, and this is the primary reason for drug resistance incidences in patients treated with systemic therapies or IT. Therefore, subsequent progression of BrM is not uncommon [245]. The precise mechanisms for drug resistance, including IT drug resistance, are not well understood even in primary tumors, and almost no information is available for therapeutic resistance in BrM [25, 246]. An interesting study on LC BrM suggests that drug resistance is an independent event in BrM, which can develop without drug treatment [247]. The immune-suppressive TME of the brain is a major factor that limits the efficacy of IT or systemic therapies and further generates conditions of therapeutic resistance [248–250]. Recently, Niesel et al. demonstrated the higher efficacy of RT + IT (WBRT + anti-PD-1) in BC BrM, where T-cell therapies fail to show an anti-tumor immune response [250]. The BrM lesions showed the accumulation of T-cells expressing PD-1, whereas recruited myeloid cells and tumor cells showed high PD-L1 expression, and eventually, anti-PD1 monotherapy did not show efficacy and became non-responsive. RT modulated the tumor immune microenvironment (TIME), increased the infiltration of CD8^+^ T cells, and prolonged median survival in BC BrM experimental models [250]. It has been shown that the combination of RT + IT prevented the activation of compensatory inhibitory mechanism(s) responses observed in BrM cases that were non-responsive to IT (anti-PD-1) alone. The outcomes of the study suggest that RT facilitates the recruitment of myeloid cells and sensitizes BrM to IT, and it also provides a rationale to test other IT drug(s) in combination with RT and/or systemic therapies to elicit the prominent immune response in BrM patients showing acquired resistant to ITs. One study examined the failure of ICIs in patients with advanced NSCLC, of which 45% had BrM, and found that the use of combined chemotherapy (paclitaxel) with a VEGF inhibitor (bevacizumab) is an appropriate salvage therapy option [251]. The median OS after salvage therapy in patients was 10.8 months, while the median PFS was 5.7 months [251]. Interestingly the median PFS for patients treated with salvage therapy after ICI failure was significantly superior to those not treated with previous ICI, at 7.0 months versus 5.2 months, respectively [251]. However, the use of previous ICI was not associated with increased OS after salvage therapy compared to patients that had not previously received ICI therapy [251]. This study proves that while IT can fail in advanced disease, its previous use is still beneficial to patients treated with subsequent salvage therapy.

## Concluding remarks and the future of immunotherapy in brain metastases

It is evident that IT is evolving as a promising approach to treating BrM from various primary tumors. Multiple immunotherapeutic drugs have been evaluated and are currently under active clinical trials **(**Tables 1 and 2**)**. However, the vulnerabilities of BrM developed from specific tumor types towards a specific immune monotherapy or combined with radiation and/or chemotherapy or salvage therapy remain to be translated. The barriers to treating BrM tumors are different compared to primary tumor types showing frequent BrM, including melanoma, breast, colorectal, renal, and lung cancer as the brain has additional protective layers for any foreign molecule or invaders, and one of them is the BBB, with the second being the immune privileged microenvironment, which might not be the case in most primary tumors [25, 247].

As far as the drug delivery problem is concerned, the future of IT in BrM hinges on new and exciting drug delivery systems that are more precise to sites of high tumor burden. In recent times, the use of nanoparticle-based drug delivery systems is an avenue that is vigorously being explored in various diagnostics and treatment settings [252]. One study investigated the use of tumor-targeting enGeneIC dream vector (EDV) nanocells that function as IT, by delivering a cytotoxin along with immune system activation [253]. The nanocells activate natural killer cells and polarize M1 macrophages, while concurrently secreting Th1 cytokines, resulting in an antitumor immune response [253]. The nanocells induced antigen presentation and dendritic cell maturation, which generated CD8^+^ T cells specific to the tumor [253]. EDV is superior to current IT because immune cell activation occurs primarily at the tumor site [253]. It elicits the priming of multiple aspects of the immune environment and is particularly advantageous in patients showing scant tumor immune response [253]. The unique properties of these nanocells could be utilized for developing IT carriers that show high specificities towards brain cells or the microenvironment and can penetrate the BBB.

Another potential avenue is drug repurposing, a strategy for identifying and employing drugs that have been approved for use in one type of clinical scenario, for another disease or clinical purpose. One such study examined repurposing the pro-senescence properties of doxorubicin, a chemotherapy drug, to introduce IT in BC BrM in mouse models [254]. Doxorubicin typically works by stopping cancer cell growth by blocking the enzyme topoisomerase 2, which is essential for resolving knots in DNA by forming double-stranded breaks and then re-ligating the DNA strands. The study found that inducing senescence of BC BrM cells by liposomal-doxorubicin (which can cross the BBB) triggers the recruitment of PD-1 expressing CD8^+^ T-cells to the brain, where they can be employed to fight tumor cells aided with PD-1 inhibitors [254]. This study provides scope for further research in using doxorubicin in conjunction with ITs to widen their clinical lens in BC BrM.

Currently, several clinical trials are in progress to expand the use of IT in BrMs. One such trial is NCT03696030, a phase I study investigating the use of intraventricular introduction of HER2.CAR T-cells in the treatment of BrM and leptomeningeal spread of recurrent cancer. Several dendritic cell vaccine trials are underway for the treatment of BrM. The clinical trial (NCT04348747) aims to examine the use of dendritic cell vaccines against HER2/HER3 along pembrolizumab treatment for BrM from TNBC or HER2 + BC. Another dendritic cell vaccine phase I trial (NCT02808416) has been completed, which is unique for its use of mRNA-encoded tumor antigens to treat patients with BrM. The NCT03638765 clinical trial investigated the use of a dendritic cell vaccine for BrM from BC or LC via direct administration to BrM through the Ommaya reservoir, a ventricular access device used for the repetitive access of the intrathecal space [255]. The NCT02774291 clinical trial examined the use of Anti-ESO (Cancer/​Test Antigen) mTCR (T-cell receptor)-transduced autologous peripheral blood lymphocytes and with chemotherapy combination for treating patients with metastatic cancer that expresses New York esophageal squamous cell carcinoma 1 (NY-ESO-1), which is an immunogenic peptide generated from the cancer-testis antigen. This trial specifically examined HLA-A2 + BrM. Lastly, memory-like natural killer cells in combination with nivolumab and relatlimab are being examined in clinical trial NCT05629546 in patients with advanced or metastatic melanoma, including patients with stable BrM. Memory-like natural killer cells are more flexible and aggressively respond to tumor targets [256].

In addition, the utilization of the metabolic axis both for cancer cells and immune cells (immunometabolism) in combination with IT drug(s) is an emerging potential therapeutic approach to treat BrM. Both cancer and immune cells are highly dependent on the availability of nutrients or metabolites, such as glutamine, glucose, arginine, and asparagine [257, 258]. Some molecules, such as glucose, glutamine, or asparagine, are required for T-cell differentiation or activation. In contrast, metabolites such as lactate and kynurenine promote Treg or exhaustion of effector T cells, thus building an immunosuppressive TME **(**Fig. 4A**)** [257, 258]. The metabolic reprogramming in cancer cells utilizes most of the glutamine, tryptophan, or glucose from TME and secretes lactate and kynurenine to the TME [258, 259]. It is imperative to utilize drug(s) or small molecule inhibitors that specifically target or restrict cancer cells to utilize glutamine, tryptophan, or glucose from TME, so that these biomolecules will be available to foster the recruitment, differentiation, and activation of immune cells in the brain microenvironment. Pro-drugs targeting specific metabolism or metabolite only in cancer and leaving T cells and/or normal brain cells unscathed could be another viable option whereby T cells are activated to kill the starved brain metastatic tumor cells. In addition, the combination strategies employing the use of RT/SRC with particular IT drug(s) that specifically target the metabolism of cancer cells could be a viable therapeutic option for treating BrM **(**Figs. 3 and 4**)**. Another important but undermined axis is the role of the patient’s gut microbiota or the metabolites from the patient’s microbiota on the modulation of tumor immune response or gut-brain axis. It is well perceived now that metabolites derived from microorganisms have immense immunomodulatory effects, such as that short-chain fatty acid (butyrate and pentanoate) regulate the functioning of Tregs, CD8^+^ T cells, and modulate the anticancer immune response of cancer ITs [260–263]. Furthermore, recently it has been shown that the infusion of Ruminococcus, Eubacterium, Bacteroides, Akkermansia, and microbiome-encoded peptidoglycan help determine the response of CD19-CAR-T cancer immunotherapy and is also useful in segregating long-term responders from non-responders [263]. Nonetheless, whichever avenues are pursued for developing future immunotherapeutic modalities for targeting BrM, the desirable upshot would be a synergizing drug(s) and/or RT with current ITs to develop a personalized or TT that can elicit a favorable antitumor immune response in difficult-to-treat patients with BrM.

## Data Availability

Not applicable, all information in this review can be found in the reference list.

## Abbreviations

5-FC: 5-fluorocytosine
5-FU: 5-fluorouracil
ATCT: adoptive T-cell transfer
ADCs: antibody-drug conjugates
B7-H3: B7 homolog 3 protein
BBB: blood-brain barrier
BrM: brain metastasis
BMFS: brain metastasis-free survival
BC: breast cancer
CNS: central nervous system
CAR: chimeric antigen receptor
CRC: colorectal cancer
CT: computed tomography
cDC1s: conventional type-1 dendritic cells
CD: cytosine deaminase
CTLs: cytotoxic lymphocytes
CTLA-4: cytotoxic T-lymphocyte-associated protein 4
ECOG: Eastern Cooperative Oncology Group
EDV: enGeneIC dream vector
EGFR: Epidermal growth factor
ER: estrogen receptor
F1t3L: Fms-like tyrosine kinase receptor 3 ligand
FOXP3: forkhead box P3 protein
GKRS: gamma knife radiation surgery
GLUT1: glucose transporters
HR: hormone receptor
HER2: human epidermal growth factor receptor
ICI: immune checkpoint inhibitor
IT: immunotherapy
ISIM: in situ immunomodulation
IDO1: indoleamine 2,3-dioxygenase 1
iLPFS: intracranial Local Progression-Free Survival
IC-PFS: intracranial progression-free survival
IDH: isocitrate dehydrogenase
IV: intravenous
KRAS: kirsten rat sarcoma viral oncogene homolog
LC: lung cancer
LM: leptomeningeal metastasis
MRI: magnetic resonance imaging
MHC: major histocompatibility complex
MBrM: melanoma brain metastasis
MAGE-A3: Melanoma-associated antigen 3
MAPK: mitogen activated protein kinase
MEK: mitogen-activated protein kinase kinase
MDSCs: myeloid-derived suppressor cells
NY-ESO-1: New York esophageal squamous cell carcinoma 1
NSCLC: non-small cell lung cancer
OS: overall survival
OXPHOS: oxidative phosphorylation
pCR: pathologic complete response
ERK: extracellular signal-regulated kinase
POLE: polymeraseε
PET-CT: positron emission tomography-computed tomography
PR: progesterone receptor
PD-1: programmed cell death protein − 1
PD-L1: programmed death-ligand 1
PFS: progression free survival
PGC1α: proliferator-activated receptor gamma coactivator 1-alpha
RT: radiotherapy
RET: Rearranged during transfection
Tregs: regulatory T cells
SCLC: small cell lung cancer
SRS: stereotactic radiosurgery
RSRS: surgery/radiosurgery
TCR: T cell receptor
TT: targeted therapy
TNBC: triple negative BC
TAMs: tumor associated macrophages
TIME: tumor immune microenvironment
TMB: tumor mutational burden
TP53: tumor protein 53
TIL: tumor-infiltrating lymphocyte
VISTA: V-domain immunoglobulin suppressor of T cell activation
BRAF: V-raf murine sarcoma viral oncogene homolog B1
WBRT: whole brain radiation therapy
